# Associations between gestational age at birth and infection-related hospital admission rates during childhood in England: Population-based record linkage study

**DOI:** 10.1371/journal.pone.0257341

**Published:** 2021-09-23

**Authors:** Victoria Coathup, Claire Carson, Jennifer J. Kurinczuk, Alison J. Macfarlane, Elaine Boyle, Samantha Johnson, Stavros Petrou, Maria A. Quigley

**Affiliations:** 1 National Perinatal Epidemiology Unit (NPEU), Nuffield Department of Population Health (NDPH), University of Oxford, Oxford, United Kingdom; 2 School of Health Sciences, University of London, London, United Kingdom; 3 Department of Health Sciences, University of Leicester, Leicester, United Kingdom; 4 Nuffield Department of Primary Health Care Sciences, University of Oxford, Oxford, United Kingdom; Norwegian Institute of Public Health, NORWAY

## Abstract

**Background:**

Children born preterm (<37 completed weeks’ gestation) have a higher risk of infection-related morbidity than those born at term. However, few large, population-based studies have investigated the risk of infection in childhood across the full spectrum of gestational age. The objectives of this study were to explore the association between gestational age at birth and infection-related hospital admissions up to the age of 10 years, how infection-related hospital admission rates change throughout childhood, and whether being born small for gestational age (SGA) modifies this relationship.

**Methods and findings:**

Using a population-based, record-linkage cohort study design, birth registrations, birth notifications and hospital admissions were linked using a deterministic algorithm. The study population included all live, singleton births occurring in NHS hospitals in England from January 2005 to December 2006 (n = 1,018,136). The primary outcome was all infection-related inpatient hospital admissions from birth to 10 years of age, death or study end (March 2015). The secondary outcome was the type of infection-related hospital admission, grouped into broad categories. Generalised estimating equations were used to estimate adjusted rate ratios (aRRs) with 95% confidence intervals (CIs) for each gestational age category (<28, 28–29, 30–31, 32, 33, 34, 35, 36, 37, 38, 39, 40, 41 and 42 weeks) and the models were repeated by age at admission (<1, 1–2, 3–4, 5–6, and 7–10 years). An interaction term was included in the model to test whether SGA status modified the relationship between gestational age and infection-related hospital admissions. Gestational age was strongly associated with rates of infection-related hospital admissions throughout childhood. Whilst the relationship attenuated over time, at 7–10 years of age those born before 40 weeks gestation were still significantly higher in comparison to those born at 40 weeks. Children born <28 weeks had an aRR of 6.53 (5.91–7.22) during infancy, declining to 3.16 (2.50–3.99) at ages 7–10 years, in comparison to those born at 40 weeks; whilst in children born at 38 weeks, the aRRs were 1·24 (1.21–1.27) and 1·18 (1.13–1.23), during infancy and aged 7–10 years, respectively. SGA status modified the effect of gestational age (interaction P<0.0001), with the highest rate among the children born at <28 weeks and SGA. Finally, study findings indicated that the associations with gestational age varied by subgroup of infection. Whilst upper respiratory tract infections were the most common type of infection experienced by children in this cohort, lower respiratory tract infections (LRTIs) (<28 weeks, aRR = 10.61(9.55–11.79)) and invasive bacterial infections (<28 weeks, aRR = 6.02 (4.56–7.95)) were the most strongly associated with gestational age at birth. Of LRTIs experienced, bronchiolitis (<28 weeks, aRR = 11.86 (10.20–13.80)), and pneumonia (<28 weeks, aRR = 9.49 (7.95–11.32)) were the most common causes.

**Conclusions:**

Gestational age at birth was strongly associated with rates of infection-related hospital admissions during childhood and even children born a few weeks early remained at higher risk at 7–10 years of age. There was variation between clinical subgroups in the strength of relationships with gestational age. Effective infection prevention strategies should include focus on reducing the number and severity of LRTIs during early childhood.

## Introduction

Children born preterm (<37 completed weeks’ gestation) are typically born with an immature immune system, leaving them vulnerable to infection [1]. However, existing evidence also indicates an increased risk in those born at early term (37–38 weeks’ gestation), which represents a much larger group of children. It is therefore important that infection-related admission risk is explored in relation to the full spectrum of gestational age at birth. In particular, children born before 40 weeks’ gestation are susceptible to lower respiratory tract infections (LRTI). Whilst this relationship is well established [2–5], there are few large, population-based studies that have explored the relationship with other subgroups of infection, or infection more broadly, throughout childhood [6,7].

A number of record linkage studies conducted in Australia reported an inverse association between gestational age at birth and gastrointestinal infections [7,8], other clinical subgroups [6] and with overall infection risk [6,9]. In addition to this, one study found that gestation and sex specific birthweight and birth length z-scores were also associated with infection-related hospital admissions during childhood [6], indicating that intrauterine growth restriction (IUGR) also plays a role within this relationship and should therefore be explored in relation to gestational age and infection-related outcomes.

Results from a recent population-based record linkage study that we conducted—the TIGAR study [10]—indicated that children in England who were born preterm, and even those born a few weeks before their due date, were at a higher risk of hospital admissions throughout childhood than those born at term. Whilst the relationship decreased with time, there was an excess risk that persisted up to 7 to 10 years of age. The study also showed that the majority of excess admissions experienced by children born before 40 weeks’ of gestation were attributed to infections [10]. However, it remains unclear which types of infection are most strongly associated with gestational age at birth and how these relationships may change with increasing age among children born in England. Therefore, using the TIGAR cohort, the objectives of this study were to investigate this relationship by estimating the rates of overall infection-related hospital admissions from birth up to 10 years of age in children born in England, exploring how these rates change with age and differ between subgroups of infection, and investigating whether being born small for gestational age (SGA) modifies the relationship between gestational age and infection-related hospital admissions.

## Methods

### Study design and data sources

A population-based record linkage study was conducted by linking data from four different sources:

1. Office for National Statistics (ONS) birth registration records, which contain information regarding every live birth and stillbirth within England.
2. ONS death registration records, which contain data related to each death within England.
3. Birth notification records (previously known as NHS Numbers for Babies [NN4B]), which assign each baby born in England with a National Health Service (NHS) number at birth, and also contain information relating to the birth, recorded by the attending clinician.
4. Hospital Episode Statistics Admitted Patient Care (HES APC) records, which contain details of all inpatient hospital admissions to NHS hospitals within England. When a baby is born, two HES APC records are created: a HES delivery record for the mother and a HES birth record for the baby. Both records contain the same information as a standard inpatient HES APC record, but additional fields relating to the mother’s delivery and baby’s birth are also collected.

Birth registration, notification and death registration records were linked to HES Birth records and HES APC records (relating to all inpatient admissions during childhood) by NHS Digital, in collaboration with the ONS and City, University of London, using a six step deterministic algorithm, as part of a previous National Institute for Health Research (NIHR) funded study. A full description of the datasets, linkage algorithms and quality assurance methods has been published elsewhere [11,12]. This includes a brief overview of the algorithms used to link and quality assure the data, together with proportions of missing data for key variables used for these purposes [12].

### Study population

**Fig 1:**
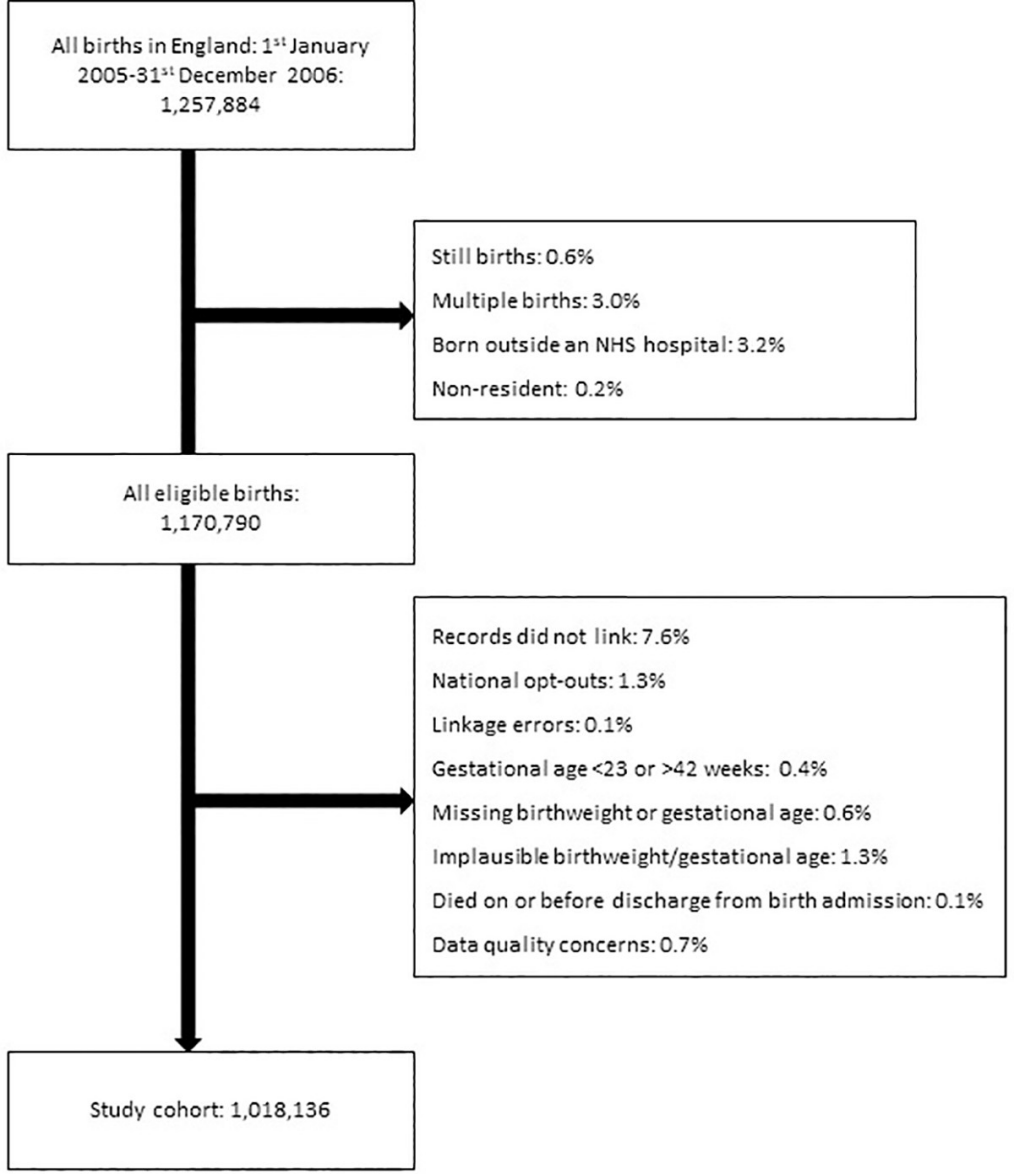
Flowchart of study cohort and exclusions.

The study population included all live, singleton births occurring within England between 1^st^ January 2005 and 31^st^ December 2006, and children were followed up from birth to 10 years of age, death or end of the study (31^st^ March 2015). A number of exclusions were made to the study cohort, including: (1) Families who had registered with the NHS national data opt-out service (a service which allows individuals to opt-out of having their patient data used for research and planning); (2) children born at home, in military or private hospitals or elsewhere (excluded as most did not have HES birth records); (3) children born to women who normally reside outside of England as it is possible these children would be admitted to hospitals in other countries during childhood; (4) children for whom gestational age or birthweight were missing; (5) children were born before 23 weeks [13] or after 42 weeks’ gestation, had a birthweight of <400g, or who had an implausible birthweight for gestational age (defined as birthweight +/- two standard deviations from the median for gestational age, sex and ethnicity) [14]; and (6) children for whom there were data quality issues. Full details can be seen in Fig 1.

### Exposures

The primary exposure of interest was gestational age at birth, which is estimated using either the date of the mother’s last menstrual period (LMP) or an ultrasound during early pregnancy and the baby’s date of birth, and was analysed in weeks using the following categories: <28, 28–29, 30–31, 32, 33, 34, 35, 36, 37, 38, 39, 40, 41, and 42 weeks, with 40 weeks selected as the reference group. Because of lower numbers of births at each gestational age at earlier gestations (23–31 weeks gestation), some weeks were combined into broader groups to ensure sufficient statistical power for estimating rate ratios. The secondary exposure of interest was small for gestational age (SGA) status. Gestation and sex specific birthweight z-scores were generated and then categorised into centiles. Children with a birthweight that was <10^th^ percentile were defined as SGA and a binary variable was created (SGA, not SGA). In models with interaction terms and in subgroups of infection with low counts, gestational age at birth was analysed using the following standard categories: <28, 28–31, 32–33, 34–36, 37–38, 39–41, and 42 weeks.

### Outcomes

The total number of infection-related hospital admissions throughout childhood was the primary outcome of interest. Infection-related hospital admissions were defined as an admission that met the following criteria:

1. The hospital admission occurred at least one day after discharge from the initial birth-related hospital admission (hospital admission records with transfer codes and ≤2 days between admission and discharge dates were considered part of the same admission). For a full list of transfer codes and further details on how a hospital admission was defined, please see S1 Table.
2. The hospital admission record contained at least one infection-related diagnosis code. Each hospital admission record contained up to 20 International Classification of Disease version 10 (ICD10) diagnosis codes and whilst the primary diagnosis code is often the main reason for admission, in practice, often the diagnosis code associated with most expensive care is listed first. Therefore, all 20 diagnosis codes were searched for relevant ICD10 codes.

Using ICD10 codes, infection-related hospital admissions were categorized into the following broad subgroups of infection, based on work from a previous study conducted in Western Australia [6]: invasive bacterial, viral, genitourinary, gastrointestinal, lower respiratory tract infections (LRTIs), upper respiratory tract infections (URTIs), and skin and soft tissue infections. Because of the strong relationship between gestational age and LRTIs, acute bronchiolitis and pneumonia were also investigated as clinical subgroups of LRTIs (for a full list of ICD10 codes, please see S1 File). Due to uncertainty around bronchiolitis diagnoses in older children, admissions for bronchiolitis occurring after one year of age were not counted [5]. If a hospital admission record contained multiple ICD10 infection codes, this record was only counted once in the main analysis looking at all infection-related hospital admissions. However, in the analyses related to specific types of infection, multiple ICD10 infection codes within any hospital admission record were not mutually exclusive, and in such cases all ICD10 codes were allocated to each infection subgroup.

Further details on how variables were derived can be seen in S1 Table.

### Statistical analysis

Key characteristics of the study population were presented as frequencies and percentages, and chi-squared tests were used to compare characteristics between those with and without at least one infection-related hospital admission during childhood. Crude hospital admission rates per 1000 person years were generated for each week of gestational age, age band (<1, 1–2, 3–4, 5–6, and 7–10 years) and for each subgroup of infection (total number of infection-related hospital admissions divided by total person time years (PTYs), which was defined as the total time from the date of discharge from the birth-related admission to death (excluding time spent in hospital with an infection), 10 years of age or study end (31^st^ March 2015), multiplied by 1000.

Due to the correlation between repeated infection-related hospital admissions, generalised estimating equations (GEE) with a negative binomial distribution and log link were used to estimate unadjusted and adjusted rate ratios (RRs) and 95% confidence intervals (CIs) for each week of gestational age. To explore whether the association between gestational age and infection-related hospital admissions was consistent over time, the model included an interaction term between gestational age (<28, 28–31, 32–33, 34–36, 37–38, 39–41, 42) and age at admission (<1, 1–2, 3–4, 5–6, 7–10). The Wald test was used to identify if this interaction was statistically significant. Negative binomial regression models were then used to estimate RRs and 95% CIs for each gestational age week within each age band. All analyses were repeated for specific subgroups of infections. To ensure sufficient statistical power to detect differences in cases where counts were low, gestational age was grouped into broader categories (<28, 28–31, 32–33, 34–36, 37–38, 39–41, and 42) with 39–41 weeks as the reference group. To assess whether being born SGA modified the relationship between gestational age and infection-related admissions, an interaction term was included between gestational age at birth and SGA status. The Wald test was used to assess the significance of the interactions and a stratified analysis was performed where deemed appropriate.

All models were adjusted for the following covariates, which were chosen *a priori* based on existing literature: maternal age at time of baby’s birth [15] (<20, 20–24, 25–29, 30–34, 35–39, ≥40 years); marital status at birth registration [16] (married, partner, single); area deprivation based on quintiles of Index of Multiple Deprivation (IMD) score [17,18] analysed in quintiles; ethnicity (white British; white other; Bangladeshi; Indian; Pakistani; black African; black Caribbean; other); mother’s country of birth [19] (UK or non-UK born); mode of birth [8] (vaginal, caesarean section); parity [2,20] (nulliparous, parous); month of birth [21] (Jan-Mar, Apr-Jun, Jul-Sept, Oct-Dec), child sex [18] (male, female) and SGA [2] (yes, no). Due to missing data in covariates, particularly ethnicity, delivery method and parity data, multiple models were developed that adjusted for groups of covariates in stages to explore how estimates were impacted.

A complete case analysis (CCA) was conducted as it was likely that some of the data was missing not at random (MNAR). For example, until May 2012 parity was only reported for births within marriage on the birth registration record, therefore missing parity was due to being unmarried. A number of strategies were undertaken to recover missing parity, which have been described in S1 Table and further detail has been published elsewhere [10]. The characteristics of those excluded from the CCA were similar to those included with the exception of mothers place of birth, marital status and child ethnicity, which differed by more than 1%. Of those excluded, mothers were less likely to be born in the UK, more likely to be married, less likely to be ‘white, British’ and more likely to be classified as the following: ‘black, British’; ‘white, other’; and ‘other’ (S2 Table).

To explore the stability of estimates, a number of sensitivity analyses were performed:

1. Children with a congenital anomaly were excluded. A congenital anomaly was defined as a relevant ICD10 code (Q) recorded in any position of diagnosis code (1–20). To exclude minor congenital anomalies that may not impact a child’s health, only those diagnosed within the first year of life were counted.
2. The relationship between gestational age and birthweight was explored in more depth by: (a) adjusting models for gestation and sex specific z-scores instead of SGA; and (b) including an interaction term between gestational age and birthweight centiles (<10^th^, 10-<25^th^, 25^th^-75^th^, 75^th^-90^th^, >90^th^).
3. Infection-related admissions that occurred within a seven-day period were counted as one admission.
4. Infection-related admissions were defined searching the primary diagnosis code only.
5. Included an interaction between gestational age and sex to explore whether child sex modified the relationship between gestational age and infection-related hospital admission rates.

All analyses were conducted using Stata 14 [22] within the Secure Research Service (SRS) of the ONS.

## Results

A total of 1,179,970 eligible births were identified. After excluding those that did not link, belonged to national opt-outs or did not meet quality standards, 1,018,136 (86%) births remained for analysis [10] with a total of 9,366,246 years of follow up. Those not included in the analysis were more likely to be babies of white ethnic origin, born outside marriage, born in particular parts of the England, or born in March, compared with those included, although these differences were small [12]. A total of 2,165 children died during follow up, with 1,203 dying during infancy, 561 between 1–2 years, 246 between 3–4 years and 155 between 7–10 years of age. There were 473,041 infection-related hospital admissions from birth up to 10 years of age and a total of 281,944 (27.7%) children experienced at least one infection-related hospital admission from birth up to 10 years of age. A total of 736,194 (72.3%) children experienced no infection-related admissions, 184,571 (18.1%) experienced one, 57,649 (5.7%) experienced two, 21,181 (2.1%) experienced three, 8,652 (0.8%) experienced four and 9,889 (1%) experienced five or more infection-related hospital admissions from birth up to 10 years of age.

Table 1 presents the key characteristics of participants. Children with at least one infection-related hospital admission were more likely to have been born to mothers who were younger, nulliparous, born in the UK, living in socially deprived areas, and unmarried; these children who were male, white, British ethnic origin, had been born after an induced labour, caesarean birth, were born SGA or who had a longer length of stay (LOS) during the birth admission were more likely to experience at least one infection-related hospital admission during childhood.

**Table 1:** Characteristics of sample population (n = 1,018,136).

	No infection-related admission (n = 736,192)	≥1 infection-related admission (n = 281,944)
	n (%)	n (%)
Socio-demographic characteristics		
Mother’s age at delivery		
*<20*	29,226 (4.0)	15,260 (5.4)
*20–24*	122,578 (16.7)	59,055 (20.9)
*25–29*	179,900 (24.4)	73,155 (25.9)
*30–34*	218,270 (29.6)	75,471 (26.8)
*35–39*	146,749 (19.9)	46,873 (16.6)
*40+*	39,469 (5.4)	12,130 (4.3)
Mother’s country of birth		
*UK*	560,831 (76.2)	230,181 (81.6)
*Non-UK*	174,241 (23.7)	51,454 (18.2)
*Missing*	1,120 (0.2)	309 (0.1)
IMD score (quintiles)		
*Q1 (most deprived)*	191,059 (26.0)	85,779 (30.4)
*Q2*	155,430 (21.1)	60,576 (21.5)
*Q3*	131,867 (17.9)	48,433 (17.2)
*Q4*	119,706 (16.3)	42,087 (14.9)
*Q5 (least deprived)*	118,861 (16.1)	38,334 (13.6)
*Missing*	19,269 (2.6)	6,735 (2.4)
Registration status		
*Married*	431,198 (58.6)	149,962 (53.2)
*Partner*	257,237 (34.9)	109,783 (38.9)
*Single*	47,757 (6.5)	22,199 (7.9)
Birth-related characteristics		
Parity		
*Nulliparous*	343,002 (46.6)	137,614 (48.8)
*Parous*	362,354 (49.2)	133,849 (47.5)
*Missing*	30,836 (4.2)	10,481 (3.7)
Delivery Method		
*Vaginal*	547,120 (74.3)	204,533 (72.5)
*C-section*	156,465 (21.3)	66,150 (23.5)
*Missing*	32,607 (4.4)	11,261 (4.0)
Season of birth		
*Jan-Mar*	172,284 (23.4)	64,660 (22.9)
*Apr-Jun*	184,469 (25.1)	69,547 (24.7)
*Jul-Sep*	195,165 (26.5)	75,117 (26.6)
*Oct-Dec*	184,274 (25.0)	72,620 (25.8)
Birth admission LOS		
*<1 week*	709,620 (96.4)	260,448 (92.4)
*1–2 weeks*	16,829 (2.3)	10,356 (3.7)
*3–4 weeks*	6,261 (0.9)	5,642 (2.0)
*1–2 months*	2,552 (0.3)	3,116 (1.1)
*3+ months*	930 (0.1)	2,382 (0.8)
Child-related characteristics		
Sex		
*Male*	365,327 (49.6)	155,842 (55.3)
*Female*	370,865 (50.4)	126,102 (44.7)
Ethnicity		
*White British*	468,816 (63.7)	208,420 (73.9)
*White Other*	46,616 (6.3)	13,067 (4.6)
*Bangladeshi*	10,430 (1.4)	4,116 (1.5)
*Indian*	20,904 (2.8)	6,879 (2.4)
*Pakistani*	28,879 (3.9)	12,860 (4.6)
*Black African*	27,439 (3.7)	7,132 (2.5)
*Black Caribbean*	9,933 (1.3)	2,477 (0.9)
*Other*	68,330 (9.3)	23,240 (8.2)
*Missing*	54,845 (7.4)	3,753 (1.3)
Gestational age (weeks)		
*<28*	471 (0.1)	1,259 (0.4)
*28–29*	805 (0.1)	1,284 (0.5)
*30–31*	1,493 (0.2)	1,734 (0.6)
*32*	1,379 (0.2)	1,277 (0.5)
*33*	2,157 (0.3)	1,893 (0.7)
*34*	4,316 (0.6)	2,976 (1.1)
*35*	7,329 (1.0)	4,334 (1.5)
*36*	15,288 (2.1)	8,058 (2.9)
*37*	36,861 (5.0)	17,140 (6.1)
*38*	97,793 (13.3)	40,133 (14.2)
*39*	168,878 (22.9)	62,498 (22.2)
*40*	212,866 (28.9)	75,199 (26.7)
*41*	155,189 (21.2)	53,568 (19.0)
*42*	31,367 (4.3)	10,591 (3.8)
Birthweight (g)		
<1500 1	252 (0.3)	3,826 (1.4)
1500–1999	5,237 (0.7)	4,244 (1.5)
2000–2499	25,013 (0.4)	13,321 (4.7)
2500–2999	122,648 (16.7)	50,459 (17.9)
3000–3499	278,649 (37.9)	102,092 (36.2)
3500–3999	224,022 (30.4)	80,143 (28.4)
4000–4499	71,050 (9.7)	25,380 (9.0)
4500+	7,046 (1.0)	2,479 (0.9)
Birthweight centiles^		
*<10* ^*th*^	71,491 (9.7)	30,638 (10.9)
*10th-<25* ^*th*^	109,829 (14.9)	42,630 (15.1)
*25* ^*th*^ *-<75* ^*th*^	369,978 (50.3)	139,490 (49.5)
*75th-90* ^*th*^	111,078 (15.1)	41,560 (14.7)
*>90* ^*th*^	73,816 (10.0)	27,626 (9.8)

*Results of chi2 tests were all p<0.0001.

^Standardised birthweight for gestational age and sex.

Crude admission rates per 100 person years were highest during infancy and for children born the most preterm. Rates declined over time, with the sharpest decline seen after the first year after birth. Children born extremely preterm (<28 weeks) had an infection-related admission rate of 1195/100 person-years during infancy. This means that if 100 children were followed up for one year, there would be 1195 infection-related hospital admissions expected during this period, indicating that some children were admitted more than once in the year (see S3 Table for the distribution of number of admissions by gestational age). By 7–10 years it was almost 48/100 person years (Table 2).

**Table 2:** Crude infection-related hospital admission rates by gestational age and age at admission.

Gestational age	N	Admissions	PTY	Rate/1000 PY
**<1 year**				
<28	1,730	1,459	1,221	1195
28–29	2,089	1,254	1,692	741
30–31	3,227	1,565	2,840	551
32	2,656	1,056	2,434	434
33	4,050	1,383	3,792	365
34	7,292	2,076	6,973	298
35	11,663	2,715	11,335	240
36	23,346	5,056	22,903	221
37	54,001	10,192	53,296	191
38	137,926	21,643	136,439	159
39	231,376	31,018	229,185	135
40	288,065	35,617	284,465	125
41	208,757	24,108	206,806	117
42	41,958	4,624	41,576	111
1–2 years				
<28	1,714	1,537	3,405	451
28–29	2,071	1,114	4,138	269
30–31	3,218	1,280	6,426	199
32	2,648	885	5,287	167
33	4,026	1,248	8,040	155
34	7,257	1,875	14,482	129
35	11,615	2,620	23,188	113
36	23,279	4,897	46,510	105
37	53,894	10,092	107,532	94
38	137,720	22,219	276,383	80
39	231,155	33,074	462,502	72
40	287,837	39,459	574,718	69
41	208,581	27,728	415,992	67
42	41,918	5,614	83,745	67
3–4 years				
<28	1,704	651	3,400	191
28–29	2,067	485	4,136	117
30–31	3,214	615	6,421	96
32	2,646	462	5,281	87
33	4,018	622	8,033	77
34	7,246	943	14,474	65
35	11,595	1,389	23,165	60
36	23,256	2,546	46,524	55
37	53,850	5,398	107,561	50
38	137,627	11,776	273,713	43
39	231,035	18,174	462,590	39
40	287,711	21,242	574,827	37
41	208,506	15,803	416,064	38
42	41,897	3,192	83,758	38
5–6 years				
<28	1,698	306	3,399	90
28–29	2,066	305	4,137	74
30–31	3,212	345	6,423	54
32	2,644	305	5,284	58
33	4,016	370	8,040	46
34	7,242	610	14,481	42
35	11,589	921	23,179	40
36	23,245	1,775	46,531	38
37	53,830	3,570	107,576	33
38	137,587	7,892	273,738	29
39	230,977	12,129	462,625	26
40	287,663	14,460	574,868	25
41	208,467	10,494	416,097	25
42	41,890	2,172	83,766	26
7–10 years				
<28	1,695	181	3,792	48
28–29	2,064	183	4,660	39
30–31	3,211	217	7,152	30
32	2,642	173	5,863	30
33	4,015	280	9,022	31
34	7,242	403	16,186	25
35	11,584	611	25,877	24
36	23,241	1,110	52,009	21
37	53,818	2,388	120,173	20
38	137,567	5,382	306,601	18
39	230,939	7,977	514,660	15
40	287,627	9,435	640,599	15
41	208,444	6,902	465,390	15
42	41,882	1,439	93,624	15

N = Total number of children, PTY = Person Time Years.

**Fig 2:**
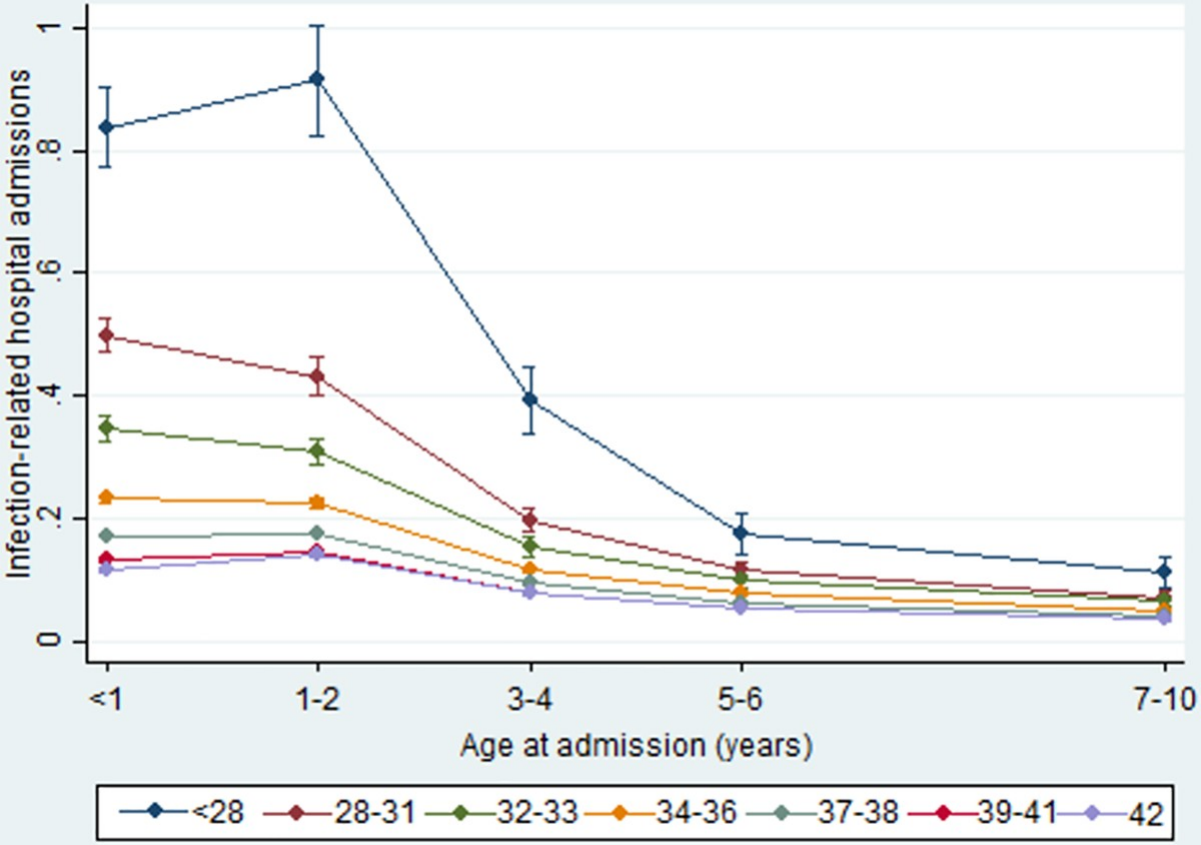
Estimated mean number of infection-related hospital admissions per child by gestational age at birth and age at admission, adjusted for: Maternal age at birth; marital status; Index of Multiple Deprivation (IMD) quintiles; ethnicity; mother’s country of birth; mode of birth; parity; month of birth, baby’s sex; and SGA.

This relationship remained in the adjusted regression models and estimates were stable in all versions of the adjusted models (S4 Table). The association between gestational age at birth and infection-related hospital admission rates was not consistent by age at admission (interaction P<0.0001). In models based on an interaction between gestational age and age, admission rates were highest during infancy and then declined over age, across all gestational ages, with the largest decrease in the most preterm groups (Fig 2).

Similarly, in models based on age-specific analyses (Table 3), there was a stronger effect of gestational age on infection-related hospital admissions at younger ages, with more than a six fold increased rate of admission during infancy compared to infants born at 40 weeks’ gestation (aRR 6.53, 95% CI 5.91–7.22). Whilst the strength of the relationship declined with age, an excess risk remained, and by 7–10 years of age adjusted rate ratios were still approximately three times higher amongst children born at <28 weeks’ gestation than for those born at 40 weeks’ gestation (aRR 3.16, 95% CI 2.50–3.99). A similar pattern was observed for children born just a few weeks early. For example, being born at 38 weeks was associated with an infection-related hospital admission rate during infancy that was 24% higher than for children born at 40 weeks’ gestation (aRR 1.24, 95% CI 1.21–1.27). Again, admission rates in this group declined over time, but by 7–10 years of age, admission rates were still almost 20% higher than those born at 40 weeks (aRR 1.18, 95% CI 1.13–1.23). Interestingly, children born post-term at 42 weeks of weeks’ gestation had a lower infection-related hospital admission rate during infancy compared to those born at 40 weeks (aRR 0.90, 95% CI 0.87–0.94), but this relationship did not persist past the first birthday. At all ages, population attributable fractions were highest for those born at 38 weeks, and the proportion of admissions attributable to being born at 37–39 weeks was 27.4% in infancy and 18.1% at age 7–10 years (S5 Table).

**Table 3:** Adjusted* rate ratios and 95% confidence intervals for infection-related hospital admissions, by gestational age at birth and age at admission.

	<1 year	1–2 years	3–4 years	5–6 years	7–10 years
Gestational age (weeks)	aRR (95% CI)	aRR (95% CI)	aRR (95% CI)	aRR (95% CI)	aRR (95% CI)
<28	6.53 (5.91, 7.22)	6.34 (5.70, 7.05)	4.94 (4.29, 5.69)	3.26 (2.73, 3.90)	3.16 (2.50, 3.99)
28–29	4.40 (3.99, 4.84)	3.58 (3.22, 3.97)	2.91 (2.53, 3.35)	2.66 (2.25, 3.15)	2.56 (2.05, 3.19)
30–31	3.47 (3.19, 3.76)	2.57 (2.35, 2.81)	2.26 (2.01, 2.55)	1.92 (1.66, 2.22)	1.78 (1.47, 2.16)
32	2.94 (2.68, 3.22)	2.19 (1.98, 2.42)	2.02 (1.77, 2.32)	2.09 (1.78, 2.44)	1.80 (1.46, 2.22)
33	2.47 (2.28, 2.67)	2.07 (1.90, 2.25)	1.90 (1.70, 2.13)	1.68 (1.47, 1.93)	1.89 (1.59, 2.24)
34	2.11 (1.98, 2.24)	1.73 (1.62, 1.85)	1.64 (1.50, 1.79)	1.59 (1.43, 1.77)	1.62 (1.42, 1.86)
35	1.74 (1.65, 1.83)	1.55 (1.46, 1.63)	1.51 (1.41, 1.63)	1.49 (1.36, 1.62)	1.53 (1.37, 1.71)
36	1.67 (1.60, 1.73)	1.47 (1.42, 1.53)	1.41 (1.34, 1.49)	1.47 (1.38, 1.57)	1.42 (1.31, 1.54)
37	1.48 (1.44, 1.52)	1.34 (1.30, 1.38)	1.32 (1.27, 1.38)	1.30 (1.24, 1.36)	1.32 (1.24, 1.40)
38	1.24 (1.21, 1.27)	1.16 (1.14, 1.19)	1.13 (1.10, 1.17)	1.13 (1.09, 1.17)	1.18 (1.13, 1.23)
39	1.08 (1.06, 1.10)	1.04 (1.02, 1.06)	1.06 (1.03, 1.09)	1.05 (1.02, 1.09)	1.04 (1.00, 1.08)
40	1.00	1.00	1.00	1.00	1.00
41	0.93 (0.91, 0.95)	0.96 (0.94, 0.97)	1.01 (0.99, 1.04)	1.00 (0.97, 1.03)	1.00 (0.96, 1.04)
42	0.90 (0.87, 0.94)	0.96 (0.93, 1.00)	1.01 (0.97, 1.06)	1.02 (0.97, 1.08)	1.07 (0.99, 1.14)

*Adjusted for: Maternal age at delivery; marital status; Index of Multiple Deprivation (IMD) quintiles; ethnicity; mother’s country of birth; mode of delivery; parity; month of birth, baby’s sex; and SGA.

**Fig 3:**
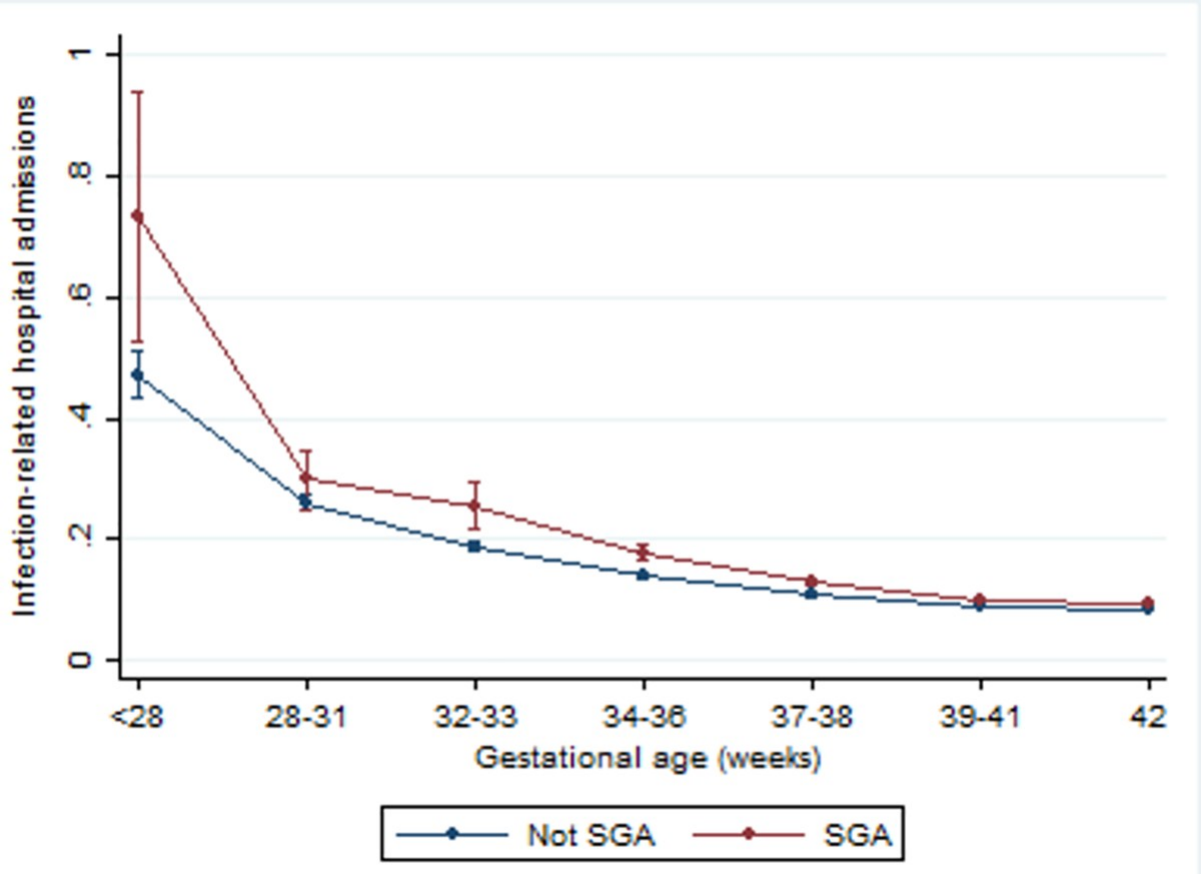
Estimated mean number of infection-related hospital admissions per child by gestational age at birth and small for gestational age (SGA), adjusted for: Maternal age at birth; marital status; Index of Multiple Deprivation (IMD) quintiles; ethnicity; mother’s country of birth; mode of birth; parity; month of birth; and baby’s sex.

There was evidence of effect modification by SGA status (interaction P<0.0001). The effect of gestational age was stronger in those born SGA compared to those not born SGA (Fig 3). When birthweight was investigated in more categories, a high birthweight (>90^th^ centile) appeared to be protective in those born extremely preterm (S1 Fig). In all sensitivity analyses, the estimates remained relatively stable (S6 Table). There was no evidence that child sex modified the relationship between gestational age and infection-related hospital admissions during childhood (interaction p = 0.110).

The 473,041 infection-related hospital admissions included 507,437 infection-related ICD10 codes, of which: 37% (187,503) were URTIs; 21.8% (110,537) were LRTIs; 10.2% (51,644) were gastro-intestinal infections; 1.6% (8,058) were invasive bacterial infections; 4.5% (22,801) were genitourinary infections; 2.8% (14,045) were skin and soft tissue infections; and 22.2% (112,849) were other viral infections. Approximately 93% of infection-related hospital admissions had only one infection-related ICD10 code. Of those records with more than one infection ICD10 code, URTI (62.7%) and viral infections (66.7%) were the most common codes to appear (S7 Table).

Crude admission rates by types of infection overall and by age at admission are presented in S1 Fig, respectively. Adjusted rate ratios were highest for those born the most preterm and were inversely associated with gestational age for all subgroups of infection (Table 4).

**Table 4:** Adjusted* rate ratios and 95% confidence intervals for gestational age at birth by type of infection, from birth to 10 years of age.

	Genitourinary	Gastrointestinal	URTI	LRTI	Other viral	Bronchiolitis^	Pneumonia
Gestational age (weeks)	aRR (95% CI)	aRR (95% CI)	aRR (95% CI)	aRR (95% CI)	aRR (95% CI)	aRR (95% CI)	aRR (95% CI)
<28	3.65 (2.62, 5.11)	2.21 (1.84, 2.66)	4.66 (4.22, 5.14)	10.61 (9.55, 11.79)	4.92 (4.40, 5.50)	11.86 (10.20, 13.80)	9.49 (7.95, 11.32)
28–29	2.70 (2.01, 3.62)	2.08 (1.56, 2.76)	2.87 (2.61, 3.15)	6.04 (5.42, 6.73)	3.29 (2.96, 3.65)	7.74 (6.68, 8.96)	6.31 (5.17, 7.70)
30–31	2.53 (1.72, 3.72)	1.78 (1.53, 2.08)	2.18 (2.03, 2.35)	4.36 (4.00, 4.75)	2.48 (2.23, 2.75)	6.31 (5.57, 7.15)	4.37 (3.75, 5.08)
32	2.40 (1.44, 4.02)	1.61 (1.36, 1.89)	2.04 (1.86, 2.24)	3.74 (3.32, 4.20)	2.14 (1.92, 2.39)	5.23 (4.53, 6.03)	3.41 (2.81, 4.15)
33	2.22 (1.76, 2.80)	1.58 (1.38, 1.80)	1.98 (1.83, 2.14)	2.98 (2.65, 3.35)	1.93 (1.77, 2.12)	3.82 (3.37, 4.34)	2.51 (2.10, 3.00)
34	2.04 (1.63, 2.54)	1.59 (1.42, 1.78)	1.61 (1.51, 1.71)	2.59 (2.38, 2.81)	1.63 (1.51, 1.75)	3.16 (2.86, 3.50)	2.42 (2.09, 2.82)
35	1.74 (1.41, 2.13)	1.30 (1.19, 1.43)	1.49 (1.41, 1.57)	2.12 (1.97, 2.28)	1.48 (1.39, 1.57)	2.43 (2.22, 2.65)	2.01 (1.76, 2.30)
36	1.57 (1.38, 1.79)	1.41 (1.32, 1.51)	1.44 (1.39, 1.50)	1.89 (1.78, 2.01)	1.42 (1.35, 1.49)	2.10 (1.96, 2.25)	1.77 (1.61, 1.95)
37	1.41 (1.28, 1.56)	1.34 (1.28, 1.41)	1.32 (1.28, 1.35)	1.62 (1.53, 1.71)	1.29 (1.24, 1.33)	1.74 (1.65, 1.83)	1.44 (1.34, 1.55)
38	1.26 (1.19, 1.34)	1.18 (1.14, 1.22)	1.15 (1.13, 1.17)	1.29 (1.25, 1.33)	1.14 (1.11, 1.17)	1.40 (1.34, 1.45)	1.24 (1.18, 1.32)
39	1.07 (1.01, 1.13)	1.06 (1.03, 1.09)	1.05 (1.03, 1.07)	1.10 (1.07, 1.13)	1.04 (1.02, 1.07)	1.14 (1.10, 1.19)	1.05 (0.99, 1.10)
40	1	1	1	1	1	1	1
41	1.00 (0.95, 1.07)	0.97 (0.94, 1.00)	0.98 (0.96, 1.00)	0.93 (0.91, 0.96)	0.97 (0.94, 0.99)	0.87 (0.84, 0.91)	0.97 (0.92, 1.03)
42	1.10 (0.97, 1.23)	0.96 (0.94, 1.00)	0.98 (0.95, 1.02)	0.92 (0.87, 0.99)	0.95 (0.91, 0.99)	0.83 (0.77, 0.90)	0.94 (0.85, 1.05)
*Adjusted for: *Maternal age at birth; marital status; Index of Multiple Deprivation (IMD) quintiles; baby’s ethnicity; mother’s country of birth; mode of birth; parity; month of birth*, *baby’s sex; and SGA*.							
*^Infections during infancy only*.							
*Note*: *Bronchiolitis and pneumonia are subgroups of the LRTI category*.							

	Invasive bacterial	Skin and soft tissue
**Gestational age (weeks)**	aRR (95% CI)	aRR (95% CI)
<28	6.02 (4.56, 7.95)	2.32 (1.68, 3.20)
28–31	3.47 (2.64, 4.56)	1.31 (1.01, 1.69)
32–33	2.38 (1.90, 2.96)	1.25 (0.97, 1.61)
34–36	1.79 (1.59, 2.01)	1.15 (1.02, 1.30)
37–38	1.27 (1.17, 1.37)	1.11 (1.05, 1.17)
39–41	1.00	1.00
42	1.04 (0.89, 1.22)	1.01 (0.90, 1.14)
*Adjusted for: *Maternal age at birth; marital status; Index of Multiple Deprivation (IMD) quintiles; baby’s ethnicity; mother’s country of birth; mode of birth; parity; month of birth*, *baby’s sex; and SGA*.		
A = URTI, B = LRTI, D = Other viral.		

**Fig 4:**
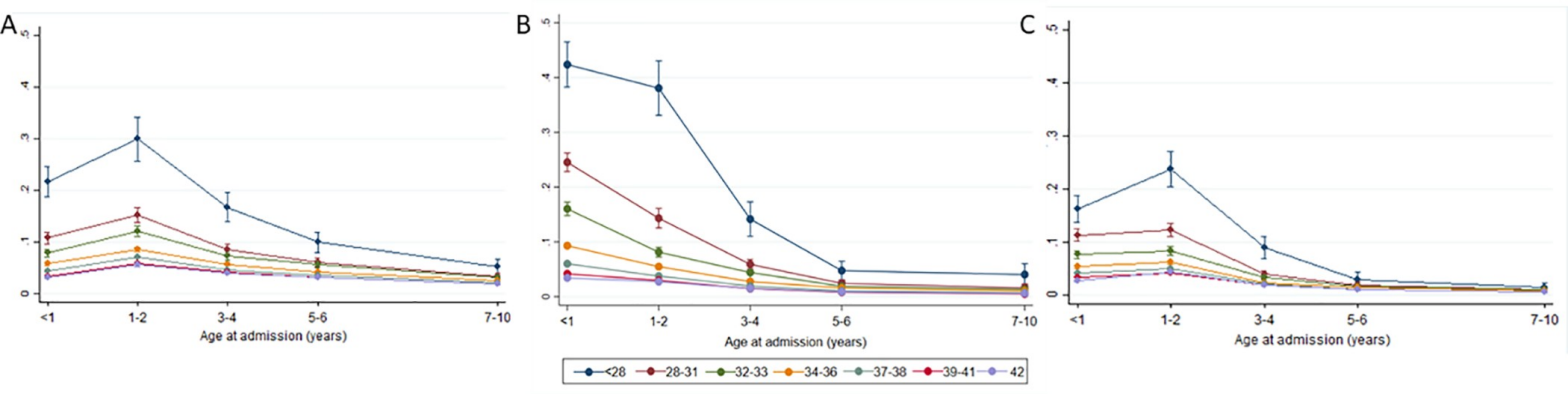
Estimated mean number of hospital admissions per child by gestational age at birth and age at admission by infection subgroups, adjusted for: Maternal age at birth; marital status; Index of Multiple Deprivation (IMD) quintiles; baby’s ethnicity; mother’s country of birth; mode of birth; parity; month of birth, baby’s sex; and SGA.

Rates of infection-related admissions for genitourinary, gastrointestinal, skin and soft tissue, and invasive bacterial infections decreased over time and were very low later in childhood. Models with interaction terms indicated that the effect of gestational age was not consistent across age groups for the three most common types of infection: LRTIs, URTIs, and other viral infections (Fig 4). For all groups, the effect of gestational age decreased after two years of age. Other viral infections and URTIs peaked during 1–2 years of age, whereas LRTIs peaked during infancy. Whilst the most common infection subgroup reported in this cohort was URTIs, LRTIs were most strongly associated with gestational age at birth; children born before 28 weeks had a LRTI hospital admission rate that was approximately 10 times that of children born at 40 weeks (aRR 10.46, 95% CI 9.42–11.61). The most common primary ICD10 diagnosis codes within this group were for unspecified LRTIs (J22.x), acute bronchiolitis (J21.9, J21.0) and pneumonia (J18.1, J18.9). Acute bronchiolitis and pneumonia accounted for approximately 30.3% and 19.9% of LRTI related admissions, respectively. Due to the strong association between LRTIs and gestational age, acute bronchiolitis and pneumonia were investigated as subgroups of the LRTI category (Table 4).

## Discussion

This study revealed that gestational age at birth was strongly associated with rates of infection-related hospital admissions during childhood. Adjusted rate ratios decreased with each additional week of gestational age and children born even a week or two early had higher infection-related admission rates than those born at 40 weeks’ gestation. Whilst the relationship attenuated over time, rates persisted at 7–10 years of age, rates for children born before 40 weeks of gestation were still significantly higher than those for children born at term. SGA status modified the effect of gestational age, with the highest rates of infection-related hospital admissions occurring among children born preterm and SGA. Finally, the findings indicated that the effects of gestational age varied by subgroups of infection. Whilst URTIs were the most common type of infection experienced by children in this cohort, LRTIs and invasive bacterial infections were the most strongly associated with gestational age at birth.

Whilst there are many studies exploring gestational age at birth in relation to specific infections during childhood, few have studied infection more broadly, particularly in a UK context. Three similar, large, population-based studies conducted in Australia all reported inverse associations between the risk of infection-related hospital admissions and gestational age at birth [6,7,9]. However, the rate ratios reported in the Australian study were lower, particularly for those born the most preterm. Differences in gestational age categories, inclusion criteria, follow up times, healthcare systems, and how infection-related hospital admissions are defined are likely to account for this. For example, Bentley et al (2018) excluded children born before 32 weeks’ gestation and those with a major congenital anomaly [7] and Miller et al. (2016) did not count repeat infection-related admissions within a 7 day period; instead, they were defined as the same admission.

The higher risk of infection associated with earlier gestational age at birth is most likely due to the baby’s immature immune system. Babies born preterm have deficiencies in both their innate and adaptive immune functions, and these systems may be further compromised by other factors related to preterm birth: perinatal infection is a major cause of preterm birth and is associated with increased intrauterine inflammation [23]; the use of antenatal corticosteroids is common, but may result in immune suppression of the neonate [1]; and the likelihood of vaginal birth and breastfeeding, which positively affect the infant microbiome [24,25], are less likely to occur in babies born preterm [26,27].

The rates of infection-related admission markedly decreased after two years of age. However, our findings indicated that nevertheless an excess risk remained at 7–10 years in children born before 40 weeks’ gestation. Whilst all babies, regardless of gestational age at birth, develop their adaptive immune systems throughout infancy and childhood, there is evidence that even at eight years of age children born preterm have reduced lymphocyte subpopulations [28], which may account for this persistent difference.

Whilst there was an inverse association between gestational age at birth and infection-related admissions for all types of infection, there was variation in the strength of this relationship by subgroup. Similar findings have been reported elsewhere. A large study followed 6.6 million children for one year in California and also reported increased odds of hospital admission for bacterial infections and respiratory infections, with odds increasing with each decreasing week of gestation. Interestingly, the relationship between gestational age and non-specific infections (fever, perinatal and viral) was not as clear; only late preterm babies were at higher risk of hospital admission than those born at full term [29]. A study of 488,603 children in New South Wales reported a significant increase in hospital admissions for acute gastroenteritis, with those born the most preterm at the greatest risk of admission [8]. A large, population-based study conducted in Western Australia reported similar differences in the strength of relationships between gestational age and the same types of infection investigated in this study [6].

The findings in this study also suggest a particularly strong relationship between gestational age at birth and LRTIs, with bronchiolitis accounting for the majority of these admissions during infancy. Similar findings have been reported elsewhere [2,5]. The authors of an Australian study also reported that whilst URTIs were also the most common infections in children, LRTIs had the strongest relationship with gestational age at birth [6]. A population-based study conducted in Wales also reported similar findings in relation to LRTIs. Compared to children born at full term, the authors reported hazard ratios that were almost 3.5 and 4 times higher for children born at <33 weeks’ gestation for LRTIs and acute bronchiolitis, respectively [2]. However, crude rates for children born at full term were slightly higher during infancy (9.8/100PY) than those found in this analysis for URTIs and LRTIs. This may be due to coding differences, as Paranjothy et al. (2013) included all respiratory admissions, rather than just respiratory infections, and higher levels of social deprivation across Wales [30], which is a known risk factor for respiratory infections during childhood [31,32]. Although bronchiolitis was strongly associated with gestational age, it was a common cause of admission in infancy across all gestational ages. A previous study in England reported that the burden of bronchiolitis admissions were in term infants without any other risk factors for severe infection [5].

Gestational age at birth was also found to be strongly associated with pneumonia-related hospital admissions during childhood, with the highest risk seen under the age of two, particularly for those born at <28 weeks’ gestation. Similar findings have been reported in other studies. A study conducted in Wales reported a hazard ratio for admissions related to pneumonia and influenza that was three times higher in children born <33 weeks’ gestation compared to those born between 39 and 42 weeks’ gestation.

The relationship between preterm birth and respiratory conditions is well established [5,31,33,34]. The lungs are one of the last organs to fully develop and babies born preterm are at greater risk of reduced surfactant production (substance that helps to keep alveoli open and inflated), difficulty clearing fluid from the lung at the time of birth and impaired gas exchange. Extremely preterm babies are also more likely to require mechanical ventilation at birth, which can result in further lung damage [1]. The impaired lung function, coupled with an immature immune system, makes children born before full term particularly at risk of LRTIs.

Paranjothy et al. (2013) also reported an independent effect of SGA on infection rates [2], suggesting that those born preterm and SGA experience a double jeopardy. One plausible mechanism for this relationship is that factors leading to intrauterine growth restriction (IUGR) experienced by SGA babies may impair the development of the lungs, the airways and the alveoli–making them more susceptible to infection [35]. Targeting interventions that may have some influence on birthweight, such as smoking cessation programmes [36] and clinical interventions, such as antiplatelet drugs at <16 weeks in women at risk of pre-eclampsia and progesterone therapy [37] may reduce the risk of IUGR [38] and therefore mitigate some of the harms associated with preterm birth.

### Strengths and limitations

To our knowledge this is the largest study conducted in England that investigates gestational age at birth in relation to all infection-related hospital admissions during childhood. The key strength of this study is the total population studied, which has enabled us to explore gestational age largely week-by-week, identifying significant differences in rates even in children born at 38 and 39 weeks’ gestation. In addition, the ten years of follow up data also enabled us to explore how rates differed by clinical subgroup and how they changed over the course of childhood. Finally, the use of routinely collected data means that it is largely unaffected by recall and social-desirability bias, which can often lead to issues in several epidemiological designs [39].

However, there are a number of limitations that must also be considered. There was variation in the quality and completeness of variables recorded within the different datasets. In addition to this, multiple imputation was not used as a method for dealing with missing data due to some of the data being potentially missing not at random (MNAR). This meant a complete case analysis was undertaken instead, and whilst the estimates remained stable in the fully adjusted model and in sensitivity analyses, we cannot rule out any potential bias being introduced through excluding those with missing data. Moreover, this study only captures the most severe cases of infection that require hospitalisation; it will underestimate rates of all infections in this population as we did not have access to data for primary care settings, where the majority of childhood infections will be managed. Additionally, we were unable to adjust for the following factors: maternal antibiotic use in pregnancy and underlying maternal conditions, which may have contributed to preterm labour and potentially the long term health of the child [40]; maternal smoking, which is a risk factor for both IUGR [41] and childhood respiratory infections in childhood [42]; maternal BMI which may increase the risk of childhood infections [43]; or explore the potentially mediating effect of breastfeeding, which is known to be protective against certain infections during early childhood [7,8]. Finally, we only included births occurring in NHS hospitals as births occurring in private or military hospitals or at home were excluded due to poor linkage rates with hospital admission records. However, it is unlikely to have biased estimates as almost 97% of births in England occur in an NHS hospital [12].

### Implications

Overall, the findings from this study have highlighted the need for effective infection prevention strategies to help protect children born preterm and close to full term. This is particularly relevant as the past two decades have seen increases in childhood hospital admissions in England for bronchiolitis [31], throat infections [44] and viral infections [45]. These may comprise a number of strategies, including: increased hygiene within and outside of clinical settings, particularly neonatal and paediatric hospital wards; improvements in air quality through methods to control pollution levels and improve ventilation in buildings [46]; interventions to support breastfeeding [47]; continued support to reduce tobacco exposure [48]; and the promotion of vaccination programmes. In addition to this, it is important that parents are counselled about how and when they should seek medical help for infections in their child. This is particularly important in the case of LRTIs as there is evidence supporting a link between LRTIs requiring hospitalisation during infancy and the subsequent development of chronic respiratory conditions [49,50].

Gestational age was strongly associated with LRTIs, in particular, bronchiolitis and pneumonia. Whilst there is an effective vaccination programme for bacterial pneumonia across England (pneumococcal conjugate vaccine has >90% coverage by the age of 2 [51]), there remains no effective vaccine for RSV, a common viral cause of pneumonia and bronchiolitis. However, a number of vaccines are currently in development [52], which have the potential to significantly reduce hospital admission rates due to these causes. Complications from influenza infections can also lead to pneumonia in children. Despite this, uptake of the influenza vaccine by pregnant women and in children up to 3 years of age remains relatively low, with coverage of approximately 44% in both populations [51,53].

Whilst the severity of respiratory infections may be greatest in those born extremely preterm, the burden of infection-related hospital admissions is largely due to those born early term and term. Since the beginning of the Covid-19 pandemic, hospital admissions for infectious diseases that are transmitted via respiratory droplets have declined [54]; most likely due to social distancing, increased hygiene and the use of facial masks in public indoor areas. Whilst all of these strategies are unlikely to remain long term, it remains to be seen how rates of infectious disease in children will be affected and whether some of the hygiene strategies currently employed will be maintained.

The findings from this study have highlighted the heterogeneity that exists within categories of gestational age. Future research should investigate gestational age at birth as a continuum, exploring it week-by-week. In addition to this, it remains unclear whether the high rates of hospital admission in children born most preterm are influenced by parents’ and clinicians’ perceptions of the child being more vulnerable due to prematurity [55]. Further research to evaluate the decision-making processes surrounding these admissions may identify areas available for potential change, thereby reducing burden on hospitals across England.

### Conclusions

The findings in this study have shown that preterm birth is strongly associated with severe infection-related morbidity, particularly during the first two years after birth. Those born the most preterm had the highest rates of infection-related hospital admission, but even children born a week or two before their due date were consistently at higher risk of infection-related hospital admissions during childhood than children born at term. Whilst the magnitude of harm is small for children born between 37–39 weeks gestation, they represent a large number of births worldwide each year. The findings further show that gestational age groups commonly investigated are not homogenous and there should be a move towards investigating the effects of gestational age at a granular level. Effective infection prevention strategies are particularly important during the current covid-19 pandemic and could include a focus on reducing the number and severity of LRTIs during early childhood.

## Supporting information

S1 Fig Estimated mean number of infection-related hospital admissions per child by gestational age and birthweight centiles, adjusted for maternal age at birth; marital status; Index of Multiple Deprivation (IMD) quintiles; baby’s ethnicity; mother’s country of birth; mode of birth; parity; month of birth, baby’s sex; and SGA. (DOCX)

S2 Fig Crude infection-related hospital admission rate per 1000 person years by gestational age, according to type of infection. Note: Bronchiolitis and pneumonia are subgroups of the LRTI category.
(DOCX)

S3 Fig Crude hospital admission rates/1000 person years by infection subgroup, gestational age at birth and age at admission. Note: Bronchiolitis and pneumonia are subgroups of the LRTI category.
(DOCX)

S1 Table Data sources and definitions for key variables. (DOCX)

S2 Table Characteristics of included and excluded individuals from sample population. (DOCX)

S3 Table Number of hospital admissions up to 10 years of age by gestational age (weeks). (DOCX)

S4 Table Rate ratios and 95% confidence intervals for infection-related hospital admissions from birth to 10 years of age, by gestational age at birth. (DOCX)

S5 Table Population attributable fractions for infection-related admissions by age at admission. (DOCX)

S6 Table Adjusted rate ratios and 95% confidence intervals for gestational age at birth (sensitivity analyses). (DOCX)

S7 Table The number of infection-related admissions with infection as the primary diagnosis code. (DOCX)

S8 Table The number of admissions with 1 or more infection codes. (DOCX)

S1 File Diagnosis codes and categories. (DOCX)
