# Supplementary material for: Associations between gestational age at birth and infection-related hospital admission rates during childhood in England: Population-based record linkage study
Source: PLoS One. 2021 Sep 23;16(9):e0257341. doi: 10.1371/journal.pone.0257341 (PMC8459942; doi:10.1371/journal.pone.0257341)
Supplement: S1 Fig — (DOCX) [file pone.0257341.s001.docx]

**
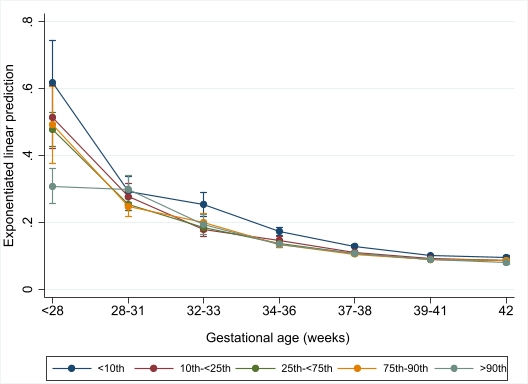
**

**Figure S1.** Estimated mean number of infection-related hospital admissions per child by gestational age and birthweight centiles, adjusted for maternal age at birth; marital status; Index of Multiple Deprivation (IMD) quintiles; baby’s ethnicity; mother’s country of birth; mode of birth; parity; month of birth, baby’s sex; and SGA
