# Supplementary material for: Associations between gestational age at birth and infection-related hospital admission rates during childhood in England: Population-based record linkage study
Source: PLoS One. 2021 Sep 23;16(9):e0257341. doi: 10.1371/journal.pone.0257341 (PMC8459942; doi:10.1371/journal.pone.0257341)
Supplement: S3 Fig — Note: Bronchiolitis and pneumonia are subgroups of the LRTI category. (DOCX) [file pone.0257341.s003.docx]

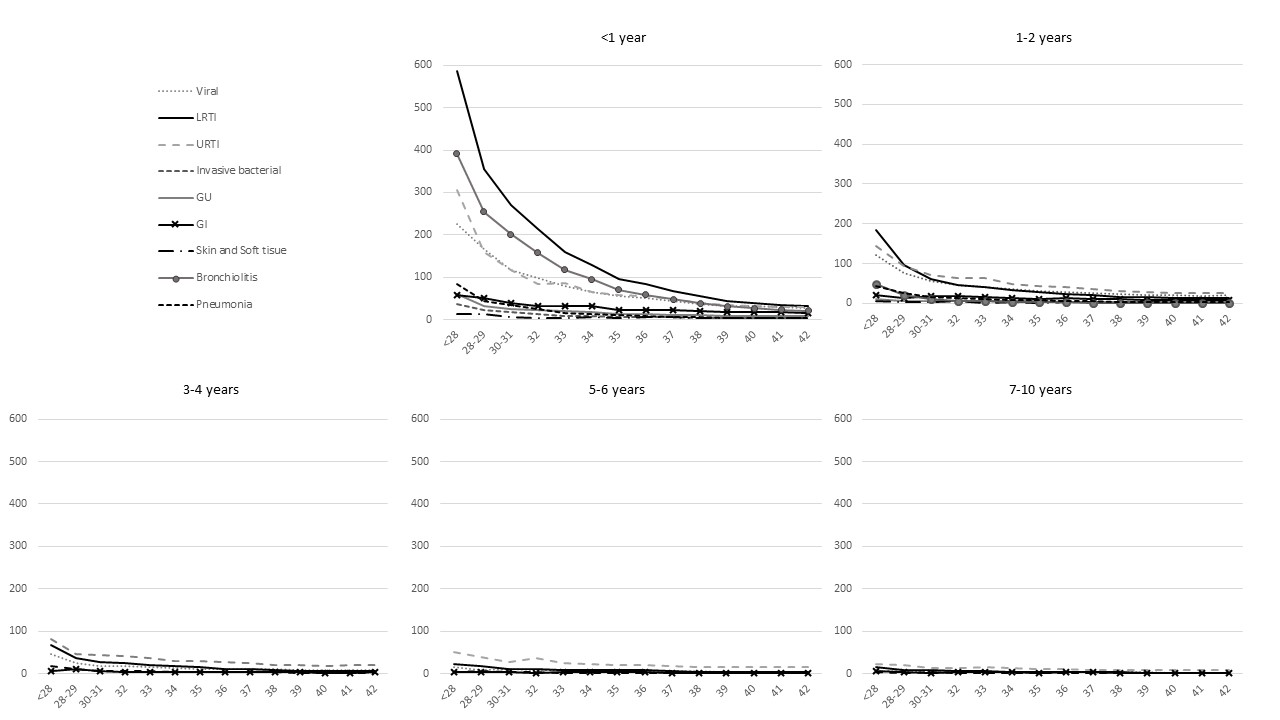


**Figure S3.** Crude hospital admission rates/1000 person years by infection subgroup, gestational age at birth and age at admission

*Note: Bronchiolitis and pneumonia are subgroups of the LRTI category*
