# Supplementary material for: Associations between gestational age at birth and infection-related hospital admission rates during childhood in England: Population-based record linkage study
Source: PLoS One. 2021 Sep 23;16(9):e0257341. doi: 10.1371/journal.pone.0257341 (PMC8459942; doi:10.1371/journal.pone.0257341)
Supplement: S1 Table — (DOCX) [file pone.0257341.s004.docx]

**Table S1.** Data sources and definitions for key variables

| Variable | Source | Definitions |
| --- | --- | --- |
| Birth-related hospital admission | HES APC | Hospital admission relating to the birth of the baby. These were identified, as they were part of Maternity HES. When a baby is born, a standard hospital in-patient admission record (HES APC) becomes a Maternity HES record. The maternity record includes information relating to the baby’s birth (these additional fields are referred to as ‘the baby tail’). Birth admission ends when infant is discharged from NHS hospital for at least one day and record contains no transfer codes. |
| Infection-related hospital admission | HES APC | Hospital admission occurring at least one day after the birth-related admission discharge, without evidence of a transfer or overlapping admission and including an infection ICD10 code (see Supplementary Information B for full list). Transfer defined as admission containing either: ADMIMETH=81; ADMISORC=49,50,51,52,53 or 87; or DISDEST=49, 50, 51, 52, 53 or 84; and ≤2 days between admission and discharge dates |
| Mother's age at delivery | Birth registration | Derived using mother's date of birth and baby's date of birth |
| IMD score | Birth registration | Derived using postcode and corresponding lower super output area (LSOA) codes. For children born in 2005, IMD score from 2004 was applied and for those born in 2006, IMD score from 2007 was applied. |
| Parity | Birth registration and HES APC | Parity only reported on the birth registration record for babies born within a marriage (PREVCHD). Therefore, a large proportion of parity was missing for all children born women who were unmarried at the time of delivery. Parity recorded in HES (NUMPREG) was either missing or poorly recorded for many women too. Therefore, Mother's HES delivery records were searched for evidence of previous deliveries: ICD10 codes = O34.2, O75.7, Z35.4, Z64.1, Z87.5 or Z87.6. For full details please see following source* |
| Mother's registration status | Birth registration | Indicator of marital status at birth registration |
| Mother's country of birth | Birth notification | Mother's country of birth field |
| Mode of delivery | HES APC | Operation codes (OPCS): R24, R23, R215, R218, R219, R211, R212, R213, R214, R22, R20, R19, R17, R18, R25. In cases with multiple delivery method codes, the most severe was chosen as delivery method. For further detail see following source* |
| Child sex | Birth registration and birth notification | Use child sex field in birth registration. If missing, use birth notification field |
| Ethnicity | Birth notification and HES APC | ETHCATZ field in birth notification used. If missing, ETHNOS in HES APC used. 17 categories mapped onto 9 categories. Ethnicity reported at notification is assumed to be child’s and not mother’s. |
| Gestational age | Birth notification | Use gestational age field from birth notification as birth registration field only reported for still births |
| Birthweight | Birth registration and birth notification | Use birthweight field from birth registration, unless field is missing or implausible, then use birth notification. For more information see following source*. Implausible birthweight for gestational age defined as + or - 2 SD from the median for gestational age, sex and ethnicity or a birthweight of <400g |
| *Macfarlane, A., Dattani, N., Gibson, R., Harper, G., Martin, P., Scanlon, M., Newburn, M., & Cortina-borja, M. (2019). Births and their outcomes by time, day and year: a retrospective birth cohort data linkage study. HEALTH SERVICES AND DELIVERY RESEARCH, 7(18). <https://doi.org/10.3310/hsdr07180> | | |
