# Supplementary material for: Associations between gestational age at birth and infection-related hospital admission rates during childhood in England: Population-based record linkage study
Source: PLoS One. 2021 Sep 23;16(9):e0257341. doi: 10.1371/journal.pone.0257341 (PMC8459942; doi:10.1371/journal.pone.0257341)
Supplement: S2 Table — (DOCX) [file pone.0257341.s005.docx]

**Table S2.** Characteristics of included and excluded individuals from sample population

|  | Full study cohort | | Complete Case analysis | | Excluded | |
| --- | --- | --- | --- | --- | --- | --- |
| Gestational age (weeks) | n | % | n | % | n | % |
| <28 | 1,730 | 0.2 | 1,518 | 0.2 | 212 | 0.2 |
| 28-29 | 2,089 | 0.2 | 1,858 | 0.2 | 231 | 0.2 |
| 30-31 | 3,227 | 0.3 | 2,862 | 0.3 | 365 | 0.3 |
| 32 | 2,656 | 0.3 | 2,351 | 0.3 | 305 | 0.2 |
| 33 | 4,050 | 0.4 | 3,618 | 0.4 | 432 | 0.3 |
| 34 | 7,292 | 0.7 | 6,436 | 0.7 | 856 | 0.7 |
| 35 | 11,663 | 1.1 | 10,250 | 1.2 | 1,412 | 1.1 |
| 36 | 23,346 | 2.3 | 20,499 | 2.3 | 2,847 | 2.2 |
| 37 | 54,001 | 5.3 | 47,285 | 5.3 | 6,716 | 5.2 |
| 38 | 137,926 | 13.5 | 120,682 | 13.6 | 17,244 | 13.4 |
| 39 | 231,376 | 22.7 | 201,765 | 22.7 | 29,611 | 23.0 |
| 40 | 288,065 | 28.3 | 251,506 | 28.3 | 36,559 | 28.4 |
| 41 | 208,757 | 20.5 | 182,420 | 20.5 | 26,337 | 20.5 |
| 42 | 41,958 | 4.1 | 36,394 | 4.1 | 5,564 | 4.3 |
| Missing | - | - | - | - | - | - |
| Birth weight (g) |  |  |  |  |  |  |
| <1500 | 6,353 | 0.6 | 5,622 | 0.6 | 731 | 0.6 |
| 1500-1999 | 9,481 | 0.9 | 8,359 | 0.9 | 1,122 | 0.9 |
| 2000-2499 | 38,334 | 3.8 | 33,372 | 3.8 | 4,962 | 3.9 |
| 2500-2999 | 173,107 | 17.0 | 150,864 | 17.0 | 22,243 | 17.3 |
| 3000-3499 | 380,741 | 37.4 | 331,563 | 37.3 | 49,178 | 38.2 |
| 3500-3999 | 304,165 | 29.9 | 266,477 | 30.0 | 37,688 | 29.3 |
| 4000-4499 | 96,430 | 9.5 | 84,750 | 9.5 | 11,680 | 9.1 |
| 4500-4999 | 9,525 | 0.9 | 8,437 | 1.0 | 1,088 | 0.8 |
| Missing | - | - | - | - | - | - |
| Mother's age (years) |  |  |  |  |  |  |
| <20 | 44,486 | 4.4 | 38,874 | 4.4 | 5,612 | 4.4 |
| 20-24 | 181,633 | 17.8 | 158,488 | 17.8 | 23,145 | 17.9 |
| 25-29 | 253,055 | 24.9 | 220,727 | 24.8 | 32,328 | 25.1 |
| 30-34 | 293,741 | 28.9 | 256,366 | 28.8 | 37,375 | 29.0 |
| 35-39 | 193,622 | 19.0 | 169,978 | 19.1 | 23,644 | 18.4 |
| 40+ | 51,599 | 5.1 | 45,011 | 5.1 | 6,588 | 5.1 |
| Mother born in UK |  |  |  |  |  |  |
| No | 225,695 | 22.2 | 187,255 | 21.1 | 38,440 | 30.2 |
| Yes | 791,012 | 77.8 | 702,189 | 78.9 | 88,823 | 69.8 |
| Missing | 1,429 | - | - |  | 1,429 | - |
| Child ethnicity |  |  |  |  |  |  |
| Bangladeshi | 15,212 | 1.6 | 13,685 | 1.5 | 1,527 | 2.3 |
| Indian | 29,887 | 3.1 | 27,895 | 3.1 | 1,992 | 3.0 |
| Pakistani | 42,710 | 4.5 | 39,442 | 4.4 | 3,268 | 5.0 |
| Black African | 34,591 | 3.6 | 29,305 | 3.3 | 5,286 | 8.1 |
| Black Caribbean | 12,412 | 1.3 | 11,111 | 1.3 | 1,301 | 2.0 |
| White British | 677,358 | 70.9 | 639,775 | 71.9 | 37,583 | 57.4 |
| White other | 59,731 | 6.3 | 54,195 | 6.1 | 5,536 | 8.5 |
| Other | 82,970 | 8.7 | 74,036 | 8.3 | 8,934 | 13.7 |
| Missing | 63,265 | - | - |  | 63,265 | - |
| Child sex |  |  |  |  |  |  |
| Male | 521,169 | 51.2 | 457,458 | 51.4 | 63,711 | 49.5 |
| Female | 496,967 | 48.8 | 431,986 | 48.6 | 64,981 | 50.5 |
| Delivery method |  |  |  |  |  |  |
| Vaginal delivery | 751,653 | 77.2 | 685,616 | 77.1 | 66,037 | 77.9 |
| C-section | 222,615 | 22.8 | 203,828 | 22.9 | 18,787 | 22.1 |
| Missing | 43,868 | - | - |  | 43,868 | - |
| Month of birth |  |  |  |  |  |  |
| Jan-Mar | 236,944 | 23.3 | 203,720 | 22.9 | 33,224 | 25.8 |
| Apr-Jun | 254,016 | 24.4 | 222,528 | 25.0 | 31,488 | 24.5 |
| Jul-Sep | 270,282 | 26.6 | 237,486 | 26.7 | 32,796 | 25.5 |
| Oct-Dec | 256,894 | 25.5 | 225,710 | 25.4 | 31,184 | 24.2 |
| Parity |  |  |  |  |  |  |
| Nulliparous | 496,203 | 50.8 | 437,300 | 49.2 | 44,059 | 50.4 |
| Parous | 480,616 | 49.2 | 452,144 | 50.8 | 43,316 | 49.6 |
| Missing | 41,317 | - | - | - | 41,317 | - |
| IMD quintiles |  |  |  |  |  |  |
| Q1 | 276,838 | 27.9 | 248,270 | 27.9 | 28,568 | 27.8 |
| Q2 | 216,006 | 21.8 | 192,893 | 21.7 | 23,113 | 22.5 |
| Q3 | 180,300 | 18.2 | 161,606 | 18.2 | 18,694 | 18.2 |
| Q4 | 161,793 | 16.3 | 144,822 | 16.3 | 16,971 | 16.5 |
| Q5 | 157,195 | 15.8 | 141,853 | 15.9 | 15,342 | 14.9 |
| Missing | 26,004 | - | - | - | 26,004 | - |
| Marital status |  |  |  |  |  |  |
| Married | 581,160 | 57.1 | 505,168 | 56.8 | 75,992 | 59.1 |
| Partner | 367,020 | 36.0 | 323,406 | 36.4 | 43,614 | 33.7 |
| Single | 69,956 | 6.9 | 60,870 | 6.8 | 9,086 | 7.1 |
