# Supplementary material for: Associations between gestational age at birth and infection-related hospital admission rates during childhood in England: Population-based record linkage study
Source: PLoS One. 2021 Sep 23;16(9):e0257341. doi: 10.1371/journal.pone.0257341 (PMC8459942; doi:10.1371/journal.pone.0257341)
Supplement: S3 Table — (DOCX) [file pone.0257341.s006.docx]

| **Table S3 .** Number of hospital admissions up to 10 years of age by gestational age (weeks) | | | | | | | | | | | | |
| --- | --- | --- | --- | --- | --- | --- | --- | --- | --- | --- | --- | --- |
|  | **0** | | **1** | | **2** | | **3** | | **4** | | **5+** | |
| **Gestational age (weeks)** | n | % | n | % | n | % | n | % | n | % | n | % |
| *<28* | 471 | 27.2 | 455 | 26.3 | 281 | 16.2 | 164 | 9.5 | 116 | 6.7 | 243 | 14.0 |
| *28-29* | 805 | 38.5 | 555 | 26.6 | 307 | 14.7 | 161 | 7.7 | 99 | 4.7 | 162 | 7.8 |
| *30-31* | 1493 | 46.3 | 833 | 25.8 | 404 | 12.5 | 223 | 6.9 | 113 | 3.5 | 161 | 5.0 |
| *32* | 1379 | 51.9 | 687 | 25.9 | 275 | 10.4 | 129 | 4.9 | 70 | 2.6 | 116 | 4.4 |
| *33* | 2,157 | 53.3 | 1034.0 | 25.5 | 438 | 10.8 | 196 | 4.8 | 89 | 2.2 | 136 | 3.4 |
| *34* | 4,316 | 59.2 | 1720.0 | 23.6 | 663 | 9.1 | 286 | 3.9 | 112 | 1.5 | 195 | 2.7 |
| *35* | 7,329 | 62.8 | 2,596 | 22.3 | 954 | 8.2 | 402 | 3.4 | 154 | 1.3 | 228 | 2.0 |
| *36* | 15,288 | 65.5 | 4,820 | 20.6 | 1,754 | 7.5 | 726 | 3.1 | 333 | 1.4 | 425 | 1.8 |
| *37* | 36,861 | 68.3 | 10,525 | 19.5 | 3,646 | 6.8 | 1,453 | 2.7 | 658 | 1.2 | 858 | 1.6 |
| *38* | 97,793 | 70.9 | 25,760 | 18.7 | 8,286 | 6.0 | 3,210 | 2.3 | 1,370 | 1.0 | 1,507 | 1.1 |
| *39* | 168,878 | 73.0 | 41,372 | 17.9 | 12,859 | 5.6 | 4,491 | 1.9 | 1,839 | 0.8 | 1,937 | 0.8 |
| *40* | 212,868 | 73.9 | 50,825 | 17.6 | 14,911 | 5.2 | 5,251 | 1.8 | 2,059 | 0.7 | 2,151 | 0.7 |
| *41* | 155,189 | 74.3 | 36,187 | 17.3 | 10,769 | 5.2 | 3,784 | 1.8 | 1,364 | 0.7 | 1,464 | 0.7 |
| *42* | 31,367 | 74.8 | 7,202 | 17.2 | 2,102 | 5.0 | 705 | 1.7 | 276 | 0.7 | 306 | 0.7 |
| **Overall** | 736,194 | 72.3 | 184,571 | 18.1 | 57,649 | 5.7 | 21,181 | 2.1 | 8,652 | 0.8 | 9,889 | 1.0 |
