# Supplementary material for: Associations between gestational age at birth and infection-related hospital admission rates during childhood in England: Population-based record linkage study
Source: PLoS One. 2021 Sep 23;16(9):e0257341. doi: 10.1371/journal.pone.0257341 (PMC8459942; doi:10.1371/journal.pone.0257341)
Supplement: S4 Table — (DOCX) [file pone.0257341.s007.docx]

**Table S4 .** Rate ratios and 95% confidence intervals for infection-related hospital admissions from birth to 10 years of age, by gestational age at birth

|  | Model 1 | Model 2 | Model 3 | Model 4 | Model 5 |
| --- | --- | --- | --- | --- | --- |
| Gestational age (weeks) | aRR (95% CI) | aRR (95% CI) | aRR (95% CI) | aRR (95% CI) | aRR (95% CI) |
| <28 | 5.81 (5.41, 6.24) | 5.81 (5.41, 6.24) | 5.84 (5.44, 6.27) | 5.66 (5.25, 6.10) | 5.47 (5.07, 5.91) |
| 28-29 | 3.87 (3.59, 4.16) | 3.84 (3.57, 4.13) | 3.81 (3.54, 4.1) | 3.61 (3.35, 3.90) | 3.49 (3.23, 3.77) |
| 30-31 | 3.00 (2.82, 3.18) | 2.97 (2.79, 3.15) | 2.95 (2.54, 3.13) | 2.77 (2.61, 2.94) | 2.63 (2.47, 2.79) |
| 32 | 2.61 (2.42, 2.82) | 2.57 (2.38, 2.77) | 2.55 (2.36, 2.75) | 2.43 (2.25, 2.620) | 2.34 (2.17, 2.53) |
| 33 | 2.33 (2.19, 2.47) | 2.29 (2.16, 2.44) | 2.27 (2.14, 2.41) | 2.17 (2.04, 2.31) | 2.10 (1.97, 2.23) |
| 34 | 1.95 (1.86, 2.05) | 1.94 (1.85, 2.03) | 1.92 (1.83, 2.01) | 1.86 (1.77, 1.95) | 1.80 (1.72, 1.90) |
| 35 | 1.71 (1.64, 1.78) | 1.7 (1.63, 1.77) | 1.69 (1.62, 1.76) | 1.63 (1.56, 1.70) | 1.60 (1.53, 1.67) |
| 36 | 1.58 (1.53, 1.64) | 1.58 (1.53, 1.63) | 1.57 (1.52, 1.62) | 1.54 (1.5, 1.60) | 1.52 (1.47, 1.57) |
| 37 | 1.41 (1.38, 1.44) | 1.41 (1.38, 1.45) | 1.42 (1.38, 1.45) | 1.39 (1.36, 1.43) | 1.38 (1.34, 1.41) |
| 38 | 1.20 (1.18, 1.22) | 1.22 (1.2, 1.24) | 1.23 (1.21, 1.24) | 1.19 (1.17, 1.21) | 1.18 (1.16, 1.20) |
| 39 | 1.06 (1.05, 1.08) | 1.07 (1.06, 1.08) | 1.08 (1.06, 1.09) | 1.06 (1.04, 1.07) | 1.06 (1.04, 1.07) |
| 40 | 1.00 | 1.00 | 1.00 | 1.00 | 1.00 |
| 41 | 0.98 (0.96, 0.99) | 0.98 (0.96, 0.99) | 0.97 (0.96, 0.98) | 0.96 (0.95, 0.98) | 0.97 (0.95, 0.98) |
| 42 | 0.97 (0.95, 1.00) | 0.97 (0.94, 1) | 0.97 (0.95, 1.00) | 0.96 (0.93, 0.99) | 0.97 (0.94, 1.00) |

*Model 1: Unadjusted*

*Model 2: Sex, mother’s age, SGA and month of birth*

*Model 3: Model 2 + Mother’s country of birth and marital status*

*Model 4: Model 3 + delivery method and parity*

*Model 5: Model 4 + ethnicity and IMD score (quintiles)*
