# Supplementary material for: Associations between gestational age at birth and infection-related hospital admission rates during childhood in England: Population-based record linkage study
Source: PLoS One. 2021 Sep 23;16(9):e0257341. doi: 10.1371/journal.pone.0257341 (PMC8459942; doi:10.1371/journal.pone.0257341)
Supplement: S5 Table — (DOCX) [file pone.0257341.s008.docx]

**Table S5.** Population attributable fractions for infection-related admissions by age at admission

| Gestational age (weeks) | <1 | 1-2 | 3-4 | 5-6 | 7-10 |
| --- | --- | --- | --- | --- | --- |
| <28 | 3.47 (3.40, 3.53) | 3.28 (3.21, 3.34) | 2.44 (2.35, 2.53) | 1.47 (1.34, 1.57) | 1.31 (1.15, 1.44) |
| 28-29 | 2.72 (2.64, 2.79) | 2.03 (1.95, 2.11) | 1.50 (1.38, 1.60) | 1.32 (1.17, 1.44) | 1.18 (1.00, 1.33) |
| 30-31 | 3.13 (3.02, 3.22) | 1.98 (1.87, 2.09) | 1.62 91.45, 1.76) | 1.14 (0.95, 1.31) | 1.01 (0.74, 1.24) |
| 32 | 1.96 (1.86, 2.05) | 1.22 (1.11, 1.32) | 1.10 (0.95, 1.24) | 1.10 (0.93, 1.25) | 0.81 (0.58, 1.01) |
| 33 | 2.31 (2.18, 2.43) | 1.63 (1.50, 1.76) | 1.39 (1.21, 1.55) | 1.04 (0.81, 1.23) | 1.40 (1.11, 1.64) |
| 34 | 3.06 (2.88, 3.23) | 2.01 (1.82, 2.19) | 1.72 (1.47, 1.95) | 1.57 (1.27, 1.83) | 1.64 (1.26, 1.97) |
| 35 | 3.24 (3.00, 3.46) | 2.35 (2.10, 2.58) | 2.22 (1.90, 2.52) | 2.08 (1.70, 2.44) | 2.24 (1.75, 2.68) |
| 36 | 5.68 (5.35, 6.01) | 3.99 (3.64, 4.32) | 3.51 (3.04, 3.95) | 3.94 (3.40, 4.45) | 3.49 (2.79, 4.13) |
| 37 | 9.30 (8.74, 9.84) | 6.46 (5.89, 7.01) | 6.21 (5.46, 6.93) | 5.74, 4.86, 6.59) | 6.12 (4.97, 7.20) |
| 38 | 11.76 (10.71, 12.80) | 7.81 (6.76, 8.85) | 6.51 (5.09, 7.88) | 6.30 (4.64, 7.91) | 8.78 (6.67, 10.79) |
| 39 | 6.28 (4.75, 7.78) | 3.32 (1.80, 4.81) | 4.84 (2.83, 6.81) | 4.30 (1.93, 6.60) | 3.24 (0.15, 6.22) |
