# Supplementary material for: Associations between gestational age at birth and infection-related hospital admission rates during childhood in England: Population-based record linkage study
Source: PLoS One. 2021 Sep 23;16(9):e0257341. doi: 10.1371/journal.pone.0257341 (PMC8459942; doi:10.1371/journal.pone.0257341)
Supplement: S6 Table — (DOCX) [file pone.0257341.s009.docx]

**Table S6.** Adjusted rate ratios and 95% confidence intervals for gestational age at birth (sensitivity analyses)

| **Gestational age (weeks)** | Excluding congenital anomalies | Adjusted for birthweight z-scores | 7-day admission period* | Primary diagnosis code only |
| --- | --- | --- | --- | --- |
|  | aRR (95% CI) | aRR (95% CI) | aRR (95% CI) | aRR (95% CI) |
| <28 | 5.35 (4.95, 5.78) | 5.41 (5.01, 5.85) | 5.59 (5.24, 5.98) | 5.46 (5.06, 5.90) |
| 28-29 | 3.39 (3.14, 3.67) | 3.46 (3.2, 3.74) | 3.72 (3.49, 3.97) | 3.47 (7.06, 3.75) |
| 30-31 | 2.57 (2.42, 2.73) | 2.61 (2.46, 2.78) | 2.92 (2.76, 3.09) | 2.62 (9.06, 2.79) |
| 32 | 2.28 (2.11, 2.47) | 2.32 (2.15, 2.51) | 2.56 (2.38, 2.76) | 2.33 (5.06, 2.52) |
| 33 | 2.05 (1.93, 2.18) | 2.09 (1.96, 2.22) | 2.29 (2.17, 2.42) | 2.09 (2.06, 2.23) |
| 34 | 1.77 (1.68, 1.86) | 1.79 (1.70, 1.88) | 1.93 (1.84, 2.02) | 1.8 (9.06, 1.89) |
| 35 | 1.57 (1.5, 1.63) | 1.58 (1.52, 1.65) | 1.7 (1.63, 1.77) | 1.59 (7.06, 1.66) |
| 36 | 1.5 (1.45, 1.55) | 1.51 (1.46, 1.56) | 1.57 (1.52, 1.61) | 1.52 (5.06, 1.57) |
| 37 | 1.37 (1.34, 1.41) | 1.37 (1.33, 1.4) | 1.39 (1.36, 1.43) | 1.37 (3.06, 1.41) |
| 38 | 1.18 (1.16, 1.2) | 1.17 (1.15, 1.19) | 1.19 (1.17, 1.21) | 1.18 (1.06, 1.2) |
| 39 | 1.06 (1.04, 1.07) | 1.05 (1.04, 1.07) | 1.06 (1.04, 1.07) | 1.06 (0.06, 1.07) |
| 40 | 1.00 | 1.00 | 1.00 | 1.00 |
| 41 | 0.97 (0.95, 0.98) | 0.97 (0.95, 0.98) | 0.98 (0.96, 0.99) | 0.97 (9.06, 0.98) |
| 42 | 0.96 (0.94, 0.99) | 0.97 (0.94, 1.00) | 0.97 (0.95, 1.00) | 0.97 (9.06, 1.00) |

**Infection-related admissions within seven days counted as one admission*
