# Supplementary material for: Associations between gestational age at birth and infection-related hospital admission rates during childhood in England: Population-based record linkage study
Source: PLoS One. 2021 Sep 23;16(9):e0257341. doi: 10.1371/journal.pone.0257341 (PMC8459942; doi:10.1371/journal.pone.0257341)
Supplement: S7 Table — (DOCX) [file pone.0257341.s010.docx]

**Table S7.** The number of infection-related admissions with infection as the primary diagnosis code

|  | n | % |
| --- | --- | --- |
| All infection | 399938 | 84.6 |
| LRTI | 97589 | 88.3 |
| URTI | 154288 | 82.3 |
| Invasive bacterial | 5391 | 67 |
| Gastro-intestinal | 46005 | 89.1 |
| Genitourinary | 17911 | 78.6 |
| Skin and soft tissue | 10371 | 73.9 |
| Viral | 68653 | 60.8 |
