# Supplementary material for: Associations between gestational age at birth and infection-related hospital admission rates during childhood in England: Population-based record linkage study
Source: PLoS One. 2021 Sep 23;16(9):e0257341. doi: 10.1371/journal.pone.0257341 (PMC8459942; doi:10.1371/journal.pone.0257341)
Supplement: S8 Table — (DOCX) [file pone.0257341.s011.docx]

**Table S8.** The number of admissions with 1 or more infection codes

|  | n | % |  |
| --- | --- | --- | --- |
| 1 code | 439961 | 93.2 |  |
| 2 codes | 31879 | 6.6 |  |
| 3 codes | 1144 | 0.2 |  |
| ≥4 codes | 60 | 0.01 |  |
