# Supplementary material for: Associations between gestational age at birth and infection-related hospital admission rates during childhood in England: Population-based record linkage study
Source: PLoS One. 2021 Sep 23;16(9):e0257341. doi: 10.1371/journal.pone.0257341 (PMC8459942; doi:10.1371/journal.pone.0257341)
Supplement: S1 File — (DOCX) [file pone.0257341.s012.docx]

**ICD10 codes: categories of infection-related hospital admission**

**Invasive bacterial**

| A02.1 Salmonella sepsis |
| --- |
| A17.0 Tuberculous meningitis |
| A17.1 Meningeal tuberculoma |
| A17.8 Other tuberculosis of nervous system |
| A17.9 Tuberculosis of nervous system, unspecified |
| A18.0 Tuberculosis of bones and joints |
| A19.0 Acute miliary tuberculosis of a single specified site |
| A19.1 Acute miliary tuberculosis of multiple sites |
| A20.3 Plague meningitis |
| A20.7 Septicemic plague |
| A21.7 Generalized tularaemia |
| A22.7 Anthrax sepsis |
| A23.0 Brucellosis due to Brucella melitensis |
| A23.1 Brucellosis due to Brucella abortus |
| A23.2 Brucellosis due to Brucella suis |
| A23.3 Brucellosis due to Brucella canis |
| A23.8 Other brucellosis |
| A23.9 Brucellosis, unspecified |
| A24.1 Acute and fulminating melioidosis |
| A25.0 Spirillosis |
| A25.1 Streptobacillosis |
| A25.9 Rat-bite fever, unspecified |
| A32.1 Listerial meningitis and meningoencephalitis |
| A32.7 Listerial sepsis |
| A39.0 Meningococcal meningitis |
| A39.1 Waterhouse-Friderichsen syndrome |
| A39.2 Acute meningococcaemia |
| A39.3 Chronic meningococcaemia |
| A39.4 Meningococcemia, unspecified |
| A39.5 Meningococcal heart disease |
| A39.8 Other meningococcal infections |
| A39.8Other meningococcal infections |
| A39.9 Meningococcal infection, unspecified |
| A40.0 Sepsis due to streptococcus, group A |
| A40.1 Sepsis due to streptococcus, group B |
| A40.2 Sepsis due to streptococcus, group D |
| A40.3 Sepsis due to Streptococcus pneumoniae |
| A40.8 Other streptococcal sepsis |
| A40.9 Streptococcal sepsis, unspecified |
| A41.0 Sepsis due to Staphylococcus aureus |
| A41.1 Sepsis due to other specified staphylococcus |
| A41.2 Sepsis due to unspecified staphylococcus |
| A41.3 Sepsis due to Hemophilus influenzae |
| A41.4 Sepsis due to anaerobes |
| A41.51 Sepsis due to Escherichia coli [E. Coli] |
| A41.52 Sepsis due to Pseudomonas |
| A41.58 Sepsis due to other Gram-negative organisms |
| A41.8 Other specified septicaemia |
| A41.9 Sepsis, unspecified |
| A44.0 Systemic bartonellosis |
| A48.3 Toxic shock syndrome |
| A52.0 Cardiovascular syphilis |
| A52.1 Symptomatic neurosyphilis |
| A52.1 (no description found) |
| A52.2 Asymptomatic neurosyphilis |
| A52.3 Neurosyphilis, unspecified |
| A52.7 Other symptomatic late syphilis |
| A52.7 (no description found) |
| A52.8 Late syphilis, latent |
| A65 Nonvenereal syphilis |
| A74.0 Chlamydial conjunctivitis |
| A74.8 Other chlamydial diseases |
| A78 Q fever |
| A79.0 Trench fever |
| A79.1 Rickettsialpox due to Rickettsia akari |
| A79.8 Other specified rickettsioses |
| A79.9 Rickettsiosis, unspecified |
| B95.1 Streptococcus, group B, as the cause of diseases classified elsewhere |
| G00.0 Haemophilus meningitis |
| G00.1 Pneumococcal meningitis |
| G00.1 (no description found) |
| G00.2 Streptococcal meningitis |
| G00.3 Staphylococcal meningitis |
| G00.8 Other bacterial meningitis |
| G00.9 Bacterial meningitis, unspecified |
| G01 Meningitis in bacterial diseases classified elsewhere |
| G03.1 Chronic meningitis |
| G05.0 Encephalitis, myelitis and encephalomyelitis in bacterial diseases classified elsewhere |
| G06.0 Intracranial abscess and granuloma |
| G06.1 Intraspinal abscess and granuloma |
| G06.2 Extradural and subdural abscess, unspecified |
| G07 Intracranial and intraspinal abscess and granuloma in disease classified elsewhere |
| I30.1 Infective pericarditis |
| I32.0 Pericarditis in bacterial diseases classified elsewhere |
| I33.0 Acute and subacute infective endocarditis |
| I41.0 Myocarditis in bacterial diseases classified elsewhere |
| J39.0 Retropharyngeal and parapharyngeal abscess |
| J39.1 Other abscess of pharynx |
| J85.3 Abscess of mediastinum |
| M00.90 Pyogenic arthritis, unspecified, multiple sites |
| M00.91 Pyogenic arthritis, unspecified, shoulder region |
| M00.92 Pyogenic arthritis, unspecified, upper arm |
| M00.93 Pyogenic arthritis, unspecified, forearm |
| M00.94 Pyogenic arthritis, unspecified, hand |
| M00.95 Pyogenic arthritis, unspecified, pelvic region and thigh |
| M00.96 Pyogenic arthritis, unspecified, lower leg |
| M00.97 Pyogenic arthritis, unspecified, ankle and foot |
| M00.98 Pyogenic arthritis, unspecified, other site |
| M00.99 Pyogenic arthritis, unspecified, site unspecified |
| M01.0 Meningococcal arthritis |
| M01.1 Tuberculous arthritis |
| M46.2 Osteomyelitis of vertebra |
| M46.3 Infection of intervertebral disc (pyogenic) |
| M46.4 Discitis, unspecified |
| M46.5 Other infective spondylopathies |
| M49.0 Tuberculosis of spine |
| M49.1 Brucella spondylitis |
| M49.2 Enterobacterial spondylitis |
| M49.3 Spondylopathy in other infectious and parasitic diseases classified elsewhere |
| M72.6 Necrotizing fasciitis |
| M73.0 Gonococcal bursitis |
| M73.1 Syphilitic bursitis |
| M86.0 Acute haematogenous osteomyelitis |
| M86.10 Other acute osteomyelitis, unspecified site |
| M86.11 Other acute osteomyelitis, shoulder region |
| M86.12 Other acute osteomyelitis, upper arm |
| M86.13 Other acute osteomyelitis, forearm |
| M86.14 Other acute osteomyelitis, hand |
| M86.15 Other acute osteomyelitis, pelvic region and thigh |
| M86.16 Other acute osteomyelitis, lower leg |
| M86.17 Other acute osteomyelitis, ankle and foot |
| M86.18 Other acute osteomyelitis, other site |
| M86.19 Other acute osteomyelitis, multiple sites |
| M86.2 Subacute osteomyelitis |
| M86.4 Chronic osteomyelitis with draining sinus |
| M86.5 Other chronic osteomyelitis |
| M86.60 Other chronic osteomyelitis, unspecified site |
| M86.67 Other chronic osteomyelitis, ankle and foot |
| M86.68 Other chronic osteomyelitis, other site |
| M86.69 Other chronic osteomyelitis, multiple sites |
| M86.8 Other osteomyelitis |
| M86.90 Unspecified osteomyelitis, multiple sites |
| M86.91 Unspecified osteomyelitis, shoulder region |
| M86.92 Unspecified osteomyelitis, upper arm |
| M86.93 Unspecified osteomyelitis, forearm |
| M86.94 Unspecified osteomyelitis, hand |
| M86.95 Unspecified osteomyelitis, pelvic region and thigh |
| M86.96 Unspecified osteomyelitis, lower leg |
| M86.97 Unspecified osteomyelitis, ankle and foot |
| M86.98 Unspecified osteomyelitis, other site |
| M86.99 Unspecified osteomyelitis, site unspecified |
| M90.0 Tuberculosis of bone |
| P36.0 Sepsis of newborn due to streptococcus, group B |
| P36.1 Sepsis of newborn due to other and unspecified streptococci |
| P36.2 Sepsis of newborn due to Staphylococcus aureus |
| P36.3 Sepsis of newborn due to other and unspecified staphylococci |
| P36.4 Sepsis of newborn due to Escherichia coli |
| P36.5 Sepsis of newborn due to anaerobes |
| P36.8 Other bacterial sepsis of newborn |
| P36.9 Bacterial sepsis of newborn, unspecified |
| P37.0 Congenital tuberculosis |
| P37.2 Neonatal (disseminated) listeriosis |
| **Gastro-intestinal infections** |
| A00.0 Cholera due to Vibrio cholerae 01, biovar cholerae |
| A00.1 Cholera due to Vibrio cholerae 01, biovar eltor |
| A00.9 Cholera, unspecified |
| A01.0 Typhoid fever |
| A01.1 Paratyphoid fever A |
| A01.2 Paratyphoid fever B |
| A01.3 Paratyphoid fever C |
| A01.4 Paratyphoid fever, unspecified |
| A02.0 Salmonella enteritis |
| A02.2 Localised salmonella infections |
| A02.8 Other specified salmonella infections |
| A02.9 Salmonella infection, unspecified |
| A03.0 Shigellosis due to Shigella dysenteriae |
| A03.1 Shigellosis due to Shigella flexneri |
| A03.2 Shigellosis due to Shigella boydii |
| A03.3 Shigellosis due to Shigella sonnei |
| A03.8 Other shigellosis |
| A03.9 Shigellosis, unspecified |
| A04.0 Enteropathogenic Escherichia coli infection |
| A04.1 Enterotoxigenic Escherichia coli infection |
| A04.2 Enteroinvasive Escherichia coli infection |
| A04.3 Enterohemorrhagic Escherichia coli infection |
| A04.4 Other intestinal Escherichia coli infections |
| A04.5 Campylobacter enteritis |
| A04.6 Enteritis due to Yersinia enterocolitica |
| A04.7 Enterocolitis due to Clostridium difficile |
| A04.8 Other specified bacterial intestinal infections |
| A04.9 Bacterial intestinal infection, unspecified |
| A05.0 Foodborne staphylococcal intoxication |
| A05.1 Botulism food poisoning |
| A05.2 Foodborne Clostridium perfringens [Clostridium welchii] intoxication |
| A05.3 Foodborne Vibrio parahaemolyticus intoxication |
| A05.4 Foodborne Bacillus cereus intoxication |
| A05.8 Other specified bacterial foodborne intoxications |
| A05.9 Bacterial foodborne intoxication, unspecified |
| A06.0 Acute amebic dysentery |
| A06.1 Chronic intestinal amebiasis |
| A06.2 Amebic nondysenteric colitis |
| A06.3 Amoeboma of intestine |
| A06.4 Amebic liver abscess |
| A06.9 Amebiasis, unspecified |
| A07.0 Balantidiasis |
| A07.1 Giardiasis [lambliasis] |
| A07.2 Cryptosporidiosis |
| A07.3 Isosporiasis |
| A07.8 Other specified protozoal intestinal diseases |
| A07.9 Protozoal intestinal disease, unspecified |
| A08.0 Rotaviral enteritis |
| A08.1 Acute gastroenteropathy due to Norwalk agent |
| A08.2 Adenoviral enteritis |
| A08.3 Other viral enteritis |
| A08.4 Viral intestinal infection, unspecified |
| A08.5 Other specified intestinal infections |
| A09 Infectious gastroenteritis and colitis, unspecified |
| A09.0 Other and unspecified gastroenteritis and colitis of infectious origin |
| A09.9 Gastroenteritis and colitis of unspecified origin |
| A21.3 Gastrointestinal tularemia |
| A22.2 Gastrointestinal anthrax |
| B37.88 Candidiasis of other sites |
| B71.0 Hymenolepiasis |
| B71.9 Cestode infection, unspecified |
| B76.0 Ancylostomiasis |
| B78.9 Strongyloidiasis, unspecified |
| B81.0 Anisakiasis |
| B96.81 Helicobacter pylori [H. pylori] as the cause of diseases classified to other chapters |
| K52.8 Other specified noninfective gastroenteritis and colitis |
| **Lower respiratory tract infections (LRTIs)** |
| A06.5 Amebic lung abscess |
| A15.0 Tuberculosis of lung |
| A15.1 Tuberculosis of lung, confirmed by culture only |
| A15.2 Tuberculosis of lung, confirmed histologically |
| A15.3 Tuberculosis of lung, confirmed by unspecified means |
| A15.4 Tuberculosis of intrathoracic lymph nodes |
| A15.5 Tuberculosis of larynx, trachea and bronchus |
| A15.6 Tuberculosis pleurisy |
| A15.7 Primary respiratory tuberculosis |
| A15.8 Other respiratory tuberculosis |
| A15.9 Respiratory tuberculosis unspecified, confirmed bacteriologically and histologically |
| A16.0 Tuberculosis of lung, bacteriologically and histologically negative |
| A16.1 Tuberculosis of lung, bacteriological and histological examination not done |
| A16.2 Tuberculosis of lung, without mention of bacteriological or histological confirmation |
| A16.3 Tuberculosis of intrathoracic lymph nodes, without mention of bacteriological or histological confirmation |
| A16.4 Tuberculosis of larynx, trachea and bronchus, without mention of bacteriological or histological confirmation |
| A16.5 Tuberculous pleurisy, without mention of bacteriological or histological confirmation |
| A16.7 Primary respiratory tuberculosis, without mention of bacteriological or histological confirmation |
| A16.8 Other respiratory tuberculosis, without mention of bacteriological or histological confirmation |
| A16.9 Respiratory tuberculosis unspecified, without mention of bacteriological or histological confirmation |
| A19.2 Acute miliary tuberculosis, unspecified |
| A19.8 Other miliary tuberculosis |
| A19.9 Miliary tuberculosis, unspecified |
| A20.2 Pneumonic plague |
| A21.2 Pulmonary tularemia |
| A22.1 Pulmonary anthrax |
| A24.0 Glanders |
| A31.0 Pulmonary mycobacterial infection |
| A37.0 Whooping cough due to Bordetella pertussis |
| A37.1 Whooping cough due to Bordetella parapertussis |
| A37.8 Whooping cough due to other Bordetella species |
| A37.9 Whooping cough, unspecified |
| A42.0 Pulmonary actinomycosis |
| A48.1 Legionnaires disease |
| A70 Chlamydia psittaci infections |
| A70 (no description found) |
| B01.2 Varicella pneumonia |
| B05.2 Measles complicated by pneumonia |
| B34.0 Adenovirus infection, unspecified |
| B37.1 Pulmonary candidiasis |
| B38.0 Acute pulmonary coccidioidomycosis |
| B38.1 Chronic pulmonary coccidioidomycosis |
| B38.2 Pulmonary coccidioidomycosis, unspecified |
| B39.0 Acute pulmonary histoplasmosis capsulati |
| B39.1 Chronic pulmonary histoplasmosis capsulati |
| B39.2 Pulmonary histoplasmosis capsulati, unspecified |
| B39.3 Disseminated histoplasmosis capsulati |
| B39.4 Histoplasmosis capsulati, unspecified |
| B39.5 Histoplasmosis duboisii |
| B39.9 Histoplasmosis, unspecified |
| B40.0 Acute pulmonary blastomycosis |
| B40.1 Chronic pulmonary blastomycosis |
| B40.2 Pulmonary blastomycosis, unspecified |
| B40.3 Cutaneous blastomycosis |
| B40.7 Disseminated blastomycosis |
| B40.8 Other forms of blastomycosis |
| B41.0 Pulmonary paracoccidioidomycosis |
| B42.0 Pulmonary sporotrichosis |
| B44.0 Invasive pulmonary aspergillosis |
| B44.1 Other pulmonary aspergillosis |
| B45.0 Pulmonary cryptococcosis |
| B58.3 Pulmonary toxoplasmosis |
| B59 Pneumocystosis |
| J05.0 Acute obstructive laryngitis [croup] |
| J09 Influenza due to certain identified influenza virus |
| J10.0 Influenza with pneumonia, influenza virus identified |
| J10.1 Influenza due to other influenza virus with respiratory manifestations |
| J11.0 Influenza with pneumonia, virus not identified |
| J11.1 Influenza with other respiratory manifestations, virus not identified |
| J12.0 Adenoviral pneumonia |
| J12.1 Respiratory syncytial virus pneumonia |
| J12.2 Parainfluenza virus pneumonia |
| J12.3 Human metapneumovirus pneumonia |
| J12.8 Other viral pneumonia |
| J12.9 Viral pneumonia, unspecified |
| J13 Pneumonia due to Streptococcus pneumoniae |
| J14 Pneumonia due to Hemophilus influenzae |
| J15.0 Pneumonia due to Klebsiella pneumoniae |
| J15.1 Pneumonia due to Pseudomonas |
| J15.2 Pneumonia due to staphylococcus |
| J15.3 Pneumonia due to streptococcus, group B |
| J15.4 Pneumonia due to other streptococci |
| J15.5 Pneumonia due to Escherichia coli |
| J15.6 Pneumonia due to other aerobic Gram-negative bacteria |
| J15.7 Pneumonia due to Mycoplasma pneumoniae |
| J15.8 Pneumonia due to other specified bacteria |
| J15.9 Unspecified bacterial pneumonia |
| J16.0 Chlamydial pneumonia |
| J16.8 Pneumonia due to other specified infectious organisms |
| J17.0 Pneumonia in bacterial diseases classified elsewhere |
| J17.1 Pneumonia in viral diseases classified elsewhere |
| J17.2 Pneumonia in mycoses |
| J17.3 Pneumonia in parasitic diseases |
| J17.8 Pneumonia in other diseases classified elsewhere |
| J18.0 Bronchopneumonia, unspecified organism |
| J18.1 Lobar pneumonia, unspecified |
| J18.8 Other pneumonia, unspecified organism |
| J18.9 Pneumonia, unspecified |
| J20.0 Acute bronchitis due to Mycoplasma pneumoniae |
| J20.1 Acute bronchitis due to Hemophilus influenzae |
| J20.2 Acute bronchitis due to streptococcus |
| J20.3 Acute bronchitis due to coxsackievirus |
| J20.4 Acute bronchitis due to parainfluenza virus |
| J20.5 Acute bronchitis due to respiratory syncytial virus |
| J20.6 Acute bronchitis due to rhinovirus |
| J20.7 Acute bronchitis due to echovirus |
| J20.8 Acute bronchitis due to other specified organisms |
| J20.9 Acute bronchitis, unspecified |
| J21.0 Acute bronchiolitis due to respiratory syncytial virus |
| J21.1 Acute bronchiolitis due to human megapneumovirus |
| J21.8 Acute bronchiolitis due to other specified organisms |
| J21.9 Acute bronchiolitis, unspecified |
| J22 Unspecified acute lower respiratory infection |
| J22.0 (no description found) |
| J40 Bronchitis, not specified as acute or chronic |
| J41.0 Simple chronic bronchitis |
| J41.1 Mucopurulent chronic bronchitis |
| J41.8 Mixed simple and mucopurulent chronic bronchitis |
| J42 Unspecified chronic bronchitis |
| J44.0 Chronic obstructive pulmonary disease with acute lower respiratory infection |
| J47 Bronchiectasis |
| J65 Pneumoconiosis associated with tuberculosis |
| J85.0 Gangrene and necrosis of lung |
| J85.1 Abscess of lung with pneumonia |
| J85.2 Abscess of lung without pneumonia |
| J86.0 Pyothorax with fistula |
| J86.9 Pyothorax without fistula |
| P23.0 Congenital pneumonia due to viral agent |
| P23.2 Congenital pneumonia due to staphylococcus |
| P23.3 Congenital pneumonia due to staphylococcus, group B |
| P23.4 Congenital pneumonia due to Escherichia coli |
| P23.5 Congenital pneumonia due to Pseudomonas |
| P23.6 Congenital pneumonia due to other bacterial agents |
| P23.8 Congenital pneumonia due to other organisms |
| P23.9 Congenital pneumonia, unspecified |
| **Upper respiratory tract infections (URTIs)** |
| A36.0 Pharyngeal diphtheria |
| A36.1 Nasopharyngeal diphtheria |
| A36.2 Laryngeal diphtheria |
| B05.3 Measles complicated by otitis media |
| H65.0 Acute serous otitis media |
| H65.1 Other acute nonsuppurative otitis media |
| H65.2 Chronic serous otitis media |
| H65.3 Chronic mucoid otitis media |
| H65.4 Other chronic nonsuppurative otitis media |
| H65.9 Nonsuppurative otitis media, unspecified |
| H66.0 Acute suppurative otitis media |
| H66.1 Chronic tubotympanic suppurative otitis media |
| H66.2 Chronic atticoantral suppurative otitis media |
| H66.3 Other chronic suppurative otitis media |
| H66.4 Suppurative otitis media, unspecified |
| H66.9 Otitis media, unspecified |
| H67.0 Otitis media in bacterial diseases classified elsewhere |
| H67.8 Otitis media in other diseases classified elsewhere |
| H68.0 Eustachian salpingitis |
| H70.0 Acute mastoiditis |
| H70.1 Chronic mastoiditis |
| H70.2 Petrositis |
| H70.8 Other mastoiditis and related conditions |
| H70.9 Mastoiditis, unspecified |
| H72.0 Central perforation of tympanic membrane |
| H72.1 Attic perforation of tympanic membrane |
| H72.2 Other marginal perforations of tympanic membrane |
| H72.8 Other perforations of tympanic membrane |
| H72.9 Perforation of tympanic membrane, unspecified |
| H73.0 Acute myringitis |
| H75.0 Mastoiditis in infectious and parasitic diseases classified elsewhere |
| H83.0 Labyrinthitis |
| H92.1 Otorrhoea |
| J00 Acute nasopharyngitis [common cold] |
| J01.0 Acute maxillary sinusitis |
| J01.1 Acute frontal sinusitis |
| J01.2 Acute ethmoidal sinusitis |
| J01.3 Acute sphenoidal sinusitis |
| J01.4 Acute pansinusitis |
| J01.8 Other acute sinusitis |
| J01.9 Acute sinusitis, unspecified |
| J02.0 Streptococcal pharyngitis |
| J02.8 Acute pharyngitis due to other specified organisms |
| J02.9 Acute pharyngitis, unspecified |
| J03.0 Streptococcal tonsillitis |
| J03.8 Acute tonsillitis due to other specified organisms |
| J03.9 Acute tonsillitis, unspecified |
| J04.0 Acute laryngitis |
| J04.1 Acute tracheitis |
| J04.2 Acute laryngotracheitis |
| J05.1 Acute epiglottitis |
| J06.0 Acute laryngopharyngitis |
| J06.8 Other acute upper respiratory infections of multiple sites |
| J06.9 Acute upper respiratory infection, unspecified |
| J32.0 Chronic maxillary sinusitis |
| J32.1 Chronic frontal sinusitis |
| J32.2 Chronic ethmoidal sinusitis |
| J32.3 Chronic sphenoidal sinusitis |
| J32.4 Chronic pansinusitis |
| J32.8 Other chronic sinusitis |
| J32.9 Chronic sinusitis, unspecified |
| J34.0 Abscess, furuncle and carbuncle of nose |
| J35.0 Chronic tonsillitis |
| J36 Peritonsillar abscess |
| **Skin and soft tissue infections** |
| A31.1 Cutaneous mycobacterial infection |
| A46 Erysipelas |
| B00.0 Eczema herpeticum |
| B35.0 Tinea barbae and tinea capitis |
| B35.1 Tinea unguium |
| B35.2 Tinea manuum |
| B35.3 Tinea pedis |
| B35.4 Tinea corporis |
| B35.5 Tinea imbricate |
| B35.6 Tinea cruris |
| B35.8 Other dermatophytoses |
| B35.9 Dermatophytosis, unspecified |
| B36.0 Pityriasis versicolour |
| B36.1 Tinea nigra |
| B36.2 White piedra |
| B36.3 Black piedra |
| B36.8 Other specified superficial mycoses |
| B37.2 Candidiasis of skin and nail |
| B85.0 Pediculosis due to Pediculus humanus capitis |
| B85.1 Pediculosis due to Pediculus humanus corporis |
| B85.2 Pediculosis, unspecified |
| B85.3 Phthiriasis |
| B85.4 Mixed pediculosis and phthiriasis |
| B86 Scabies |
| B87.9 Myiasis, unspecified |
| B88.0 Other acariasis |
| B88.1 Tungiasis [sandflea infestation] |
| B88.2 Other arthropod infestations |
| B88.3 External hirudiniasis |
| B88.8 Other specified infestations |
| B88.9 Infestation, unspecified |
| H60.3 Other infective otitis externa |
| L00 Staphylococcal scalded skin syndrome |
| L01.0 Impetigo [any organism] [any site] |
| L02.0 Cutaneous abscess, furuncle and carbuncle of face |
| L02.1 Cutaneous abscess, furuncle and carbuncle of neck |
| L02.2 Cutaneous abscess, furuncle and carbuncle of trunk |
| L02.3 Cutaneous abscess, furuncle and carbuncle of buttock |
| L02.4 Cutaneous abscess, furuncle and carbuncle of limb |
| L02.8 Cutaneous abscess, furuncle and carbuncle of other sites |
| L02.9 Cutaneous abscess, furuncle and carbuncle, unspecified |
| L03.01 Cellulitis of finger |
| L03.02 Cellulitis of toe |
| L03.10 Cellulitis of upper limb |
| L03.11 Cellulitis of lower limb |
| L03.2 Cellulitis of face |
| L03.3 Cellulitis of trunk |
| L03.8 Cellulitis of other sites |
| L03.9 Cellulitis, unspecified |
| L05.0 Pilonidal cyst with abscess |
| L05.9 Pilonidal cyst without abscess |
| L08.0 Pyoderma |
| L08.1 Erythrasma |
| L08.8 Other specified local infections of skin and subcutaneous tissue |
| L08.9 Local infection of the skin and subcutaneous tissue, unspecified |
| L13.0 Dermatitis herpetiformis |
| L30.3 Infective dermatitis |
| L88 Pyoderma gangrenosum |
| M60.09 Infective myositis, multiple sites |
| M63.0 Myositis in bacterial diseases classified elsewhere |
| M63.1 Myositis in protozoal and parasitic infections classified elsewhere |
| M63.2 Myositis in other infectious diseases classified elsewhere |
| M65.0 Abscess of tendon sheath |
| M65.1 Other infective (teno)synovitis |
| M68.0 Synovitis and tenosynovitis in bacterial diseases classified elsewhere |
| M71.0 Abscess of bursa |
| M71.1 Other infective bursitis |
| O91.00 Infection of nipple associated with childbirth, without mention of attachment difficulty |
| O91.10 Abscess of breast associated with childbirth, without mention of attachment difficulty |
| P39.0 Neonatal infective mastitis |
| P39.4 Neonatal skin infection |
| **Genitourinary infections** |
| A51.0 Primary genital syphilis |
| A51.1 Primary anal syphilis |
| A51.2 Primary syphilis of other sites |
| A51.3 Secondary syphilis of skin and mucous membranes |
| A51.4 Other secondary syphilis |
| A51.5 Early syphilis, latent |
| A51.9 Early syphilis, unspecified |
| A52.9 Late syphilis, unspecified |
| A53.0 Latent syphilis, unspecified as early or late |
| A53.9 Syphilis, unspecified |
| A54.0 Gonococcal infection of lower genitourinary tract without periurethral or accessory gland abscess |
| A54.1 Gonococcal infection of lower genitourinary tract with periurethral and accessory gland abscess |
| A54.2 Gonococcal pelviperitonitis and other gonococcal genitourinary infections |
| A54.3 Gonococcal infection of eye |
| A54.3 (no description found) |
| A54.4 Gonococcal infection of musculoskeletal system |
| A54.5 Gonococcal pharyngitis |
| A54.6 Gonococcal infection of anus and rectum |
| A54.8 Other gonococcal infections |
| A54.8 (no description found) |
| A54.9 Gonococcal infection, unspecified |
| A55 Chlamydial lymphogranuloma (venereum) |
| A56.0 Chlamydial infection of lower genitourinary tract |
| A56.1 Chlamydial infection of pelviperitoneum and other genitourinary organs |
| A56.2 Chlamydial infection of genitourinary tract, unspecified |
| A56.3 Chlamydial infection of anus and rectum |
| A56.4 Chlamydial infection of pharynx |
| A56.8 Sexually transmitted chlamydial infection of other sites |
| A57 Chancroid |
| A58 Granuloma inguinale |
| A59.0 Urogenital trichomoniasis |
| A59.0 (no description found) |
| A60.0 Herpesviral infection of genitalia and urogenital tract |
| A60.1 Herpesviral infection of perianal skin and rectum |
| A60.9 Anogenital herpesviral infection, unpsecified |
| A63.0 Anogenital (venereal) warts |
| A63.8 Other specified predominantly sexually transmitted diseases |
| A64 Unspecified sexually transmitted disease |
| B37.3 Candidiasis of vulva and vagina |
| B37.4 Candidiasis of other urogenital sites |
| N13.6 Pyonephrosis |
| N15.1 Renal and perinephric abscess |
| N30.0 Acute cystitis |
| N30.8 Other cystitis, abscess of bladder |
| N34.0 Urethral abscess |
| N35.1 Postinfective urethral stricture, not elsewhere classified |
| N39.0 Urinary tract infection, site not specified |
| N41.0 Acute prostatitis |
| N41.2 Abscess of prostate |
| N43.1 Infected hydrocele |
| N45.0 Orchitis, epididymitis and epididymo-orchitis with abscess |
| N45.9 Orchitis, epididymitis and epididymo-orchits without abscess |
| N51.2 Balanitis in diseases classified elsewhere |
| N70.0 Acute salpingitis and oophoritis |
| N73.0 Acute parametritis and pelvic cellulitis |
| N73.1 Chronic parametritis and pelvic cellulitis |
| N73.2 Unspecified parametritis and pelvic cellulitis |
| N73.3 Female acute pelvic peritonitis |
| N73.4 Female chronic pelvic peritonitis |
| N73.9 Female pelvic inflammatory disease, unspecified |
| N74.2 Female syphilitic pelvic inflammatory disease |
| N74.3 Female gonococcal pelvic inflammatory disease |
| N74.4 Female chlamydial pelvic inflammatory disease |
| N75.0 Cyst of Bartholins gland |
| N75.1 Abscess of Bartholins gland |
| N76.0 Acute vaginitis |
| N76.4 Abscess of vulva |
| O03.0 Spontaneous abortion, incomplete, complicated by genital tract and pelvic infection |
| O03.5 Spontaneous abortion, complete or unspecified, complicated by genital tract and pelvic infection |
| O08.0 Genital tract and pelvic infection following ectopic and molar pregnancy |
| O26.4 Herpes gestationis |
| O86.2 Urinary tract infection following delivery |
| O98.1 Syphilis complicating pregnancy, childbirth and the puerperium |
| O98.2 Gonorrhea complicating pregnancy, childbirth and the puerperium |
| P39.3 Neonatal urinary tract infection |
| **Other viral infections** |
| A80.1 Acute paralytic poliomyelitis, wild virus, imported |
| A80.2 Acute paralytic poliomyelitis, wild virus, indigenous |
| A80.3 Acute paralytic poliomyelitis, other and unspecified |
| A80.4 Acute nonparalytic poliomyelitis |
| A80.9 Acute poliomyelitis, unspecified |
| A81.8 Other atypical virus infections of central nervous system |
| A81.9 Atypical virus infection of central nervous system, unspecified |
| A82.0 Sylvatic rabies |
| A82.1 Urban rabies |
| A82.9 Rabies, unspecified |
| A83.0 Japanese encephalitis |
| A83.1 Western equine encephalitis |
| A83.2 Eastern equine encephalitis |
| A83.3 St Louis encephalitis |
| A83.4 Australian encephalitis |
| A83.5 California encephalitis |
| A83.6 Rocio virus disease |
| A83.8 Other mosquito-borne viral encephalitis |
| A83.9 Mosquito-borne viral encephalitis, unspecified |
| A84.0 Far Eastern tick-borne encephalitis [Russian spring-summer encephalitis] |
| A84.1 Central European tick-borne encephalitis |
| A84.8 Other tick-borne viral encephalitis |
| A84.9 Tick-borne viral encephalitis, unspecified |
| A85.0 Enteroviral encephalitis |
| A85.1 Adenoviral encephalitis |
| A85.2 Arthropod-borne viral encephalitis, unspecified |
| A85.8 Other specified viral encephalitis |
| A86 Unspecified viral encephalitis |
| A87.0 Enteroviral meningitis |
| A87.1 Adenoviral meningitis |
| A87.2 Lymphocytic choriomeningitis |
| A87.8 Other viral meningitis |
| A87.9 Viral meningitis, unspecified |
| A88.0 Enteroviral exanthematous fever |
| A88.8 Other specified viral infections of central nervous system |
| A89 Unspecified viral infection of central nervous system |
| A90 Dengue fever [classical dengue] |
| A91 Dengue haemorrhagic fever |
| A92.0 Chikungunya virus disease |
| A92.1 O’nyong-nyong fever |
| A92.2 Venezuelan equine fever |
| A92.3 West Nile virus infection |
| A92.4 Rift Valley fever |
| A92.8 Other specified mosquito-borne viral fevers |
| A92.9 Mosquito-borne viral fever, unspecified |
| A93.0 Oropouche virus disease |
| A93.2 Colorado tick fever |
| A93.8 Other specified arthropod-borne viral fevers |
| A94 Unspecified arthropod-borne viral fever |
| A95.0 Sylvatic yellow fever |
| A95.1 Urban yellow fever |
| A95.9 Yellow fever, unspecified |
| A96.0 Junin haemorrhagic fever |
| A96.1 Machupo haemorrhagic fever |
| A96.2 Lassa fever |
| A96.8 Other arenaviral hemorrhagic fevers |
| A96.9 Arenaviral haemorrhagic fever, unspecified |
| A98.0 Crimean-Congo hemorrhagic fever |
| A98.1 Omsk hemorrhagic fever |
| A98.2 Kyasanur Forest disease |
| A98.3 Marburg virus disease |
| A98.4 Ebola virus disease |
| A98.5 Hemorrhagic fever with renal syndrome |
| A98.8 Other specified viral haemorrhagic fevers |
| A99 Unspecified viral haemorrhagic fever |
| B00.1 Herpesviral vesicular dermatitis |
| B00.2 Herpesviral gingivostomatitis and pharyngotonsillitis |
| B00.3 Herpesviral meningitis |
| B00.4 Herpesviral encephalitis |
| B00.5 Herpesviral ocular disease |
| B00.7 Disseminated herpesviral disease |
| B00.8 Other forms of herpesviral infection |
| B00.9 Herpesviral infection, unspecified |
| B01.0 Varicella meningitis |
| B01.1 Varicella encephalitis |
| B01.8 Varicella with other complications |
| B01.9 Varicella without complication |
| B02.0 Zoster encephalitis |
| B02.1 Zoster meningitis |
| B02.2 Zoster with other nervous system involvement |
| B02.3 Zoster ocular disease |
| B02.7 Disseminated zoster |
| B02.8 Zoster with other complications |
| B02.9 Zoster without complications |
| B03 Smallpox |
| B04 Monkeypox |
| B05.0 Measles complicated by encephalitis |
| B05.1 Measles complicated by meningitis |
| B05.4 Measles with intestinal complications |
| B05.8 Measles with other complications |
| B05.9 Measles without complication |
| B06.0 Rubella with neurological complications |
| B06.8 Rubella with other complications |
| B06.9 Rubella without complication |
| B07 Viral warts |
| B08.0 Other orthopoxvirus infections |
| B08.1 Molluscum contagiosum |
| B08.2 Exanthema subitum [sixth disease] |
| B08.3 Erythema infectiosum [fifth disease] |
| B08.4 Enteroviral vesicular stomatitis with exanthem |
| B08.5 Enteroviral vesicular pharyngitis |
| B08.8 Other specified viral infections characterized by skin and mucous membrane lesions |
| B09 Unspecified viral infection characterized by skin and mucous membrane lesions |
| B15.0 Hepatitis A with hepatic coma |
| B15.9 Hepatitis A without hepatic coma |
| B16.0 Acute hepatitis B with delta-agent with hepatic coma |
| B16.1 Acute hepatitis B with delta-agent without hepatic coma |
| B16.2 Acute hepatitis B without delta-agent with hepatic coma |
| B16.9 Acute hepatitis B without delta-agent and without hepatic coma |
| B17.0 Acute delta-(super) infection of hepatitis B carrier |
| B17.1 Acute hepatitis C |
| B17.2 Acute hepatitis E |
| B17.8 Other specified acute viral hepatitis |
| B17.9 Acute viral hepatitis, unspecified |
| B18.0 Chronic viral hepatitis B with delta-agent |
| B18.1 Chronic viral hepatitis B without delta-agent |
| B18.2 Chronic viral hepatitis C |
| B18.8 Other chronic viral hepatitis |
| B18.9 Chronic viral hepatitis, unspecified |
| B19.0 Unspecified viral hepatitis with hepatic coma |
| B19.9 Unspecified viral hepatitis without hepatic coma |
| B20.0 HIV disease resulting in mycobacterial infection |
| B20.1 HIV disease resulting in other bacterial infections |
| B20.2 HIV disease resulting in cytomegaloviral disease |
| B20.3 HIV disease resulting in other viral infections |
| B20.4 HIV disease resulting in candidiasis |
| B20.5 HIV disease resulting in other mycoses |
| B20.6 HIV disease resulting in Pneumocystis jirovecii pneumonia |
| B20.7 HIV disease resulting in multiple infections |
| B20.8 HIV disease resulting in other infectious and parasitic diseases |
| B20.9 HIV disease resulting in unspecified infectious or parasitic |
| B21.0 HIV disease resulting in Kaposi sarcoma |
| B21.1 HIV disease resulting in Burkitt lymphoma |
| B21.2 HIV disease resulting in other types of non-Hodgkin lymphoma |
| B21.3 HIV disease resulting in other malignant neoplasms of lymphoid, haematopoietic and related tissue |
| B21.7 HIV disease resulting in multiple malignant neoplasms |
| B21.8 HIV disease resulting in other malignant neoplasms |
| B21.9 HIV disease resulting in unspecified malignant neoplasm |
| B22.0 HIV disease resulting in encephalopathy |
| B22.1 HIV disease resulting in lymphoid interstitial pneumonitis |
| B22.2 HIV disease resulting in wasting syndrome |
| B22.7 HIV disease resulting in multiple diseases classified elsewhere |
| B23.0 Acute HIV infection syndrome |
| B23.1 HIV disease resulting in (persistent) generalized lymphadenopathy |
| B23.2 HIV disease resulting in haematological and immunological abnormalities, not elsewhere classified |
| B23.8 HIV disease resulting in other specified conditions |
| B24 Unspecified human immunodeficiency virus [HIV] disease |
| B25.0 Cytomegaloviral pneumonitis |
| B25.1 Cytomegaloviral hepatitis |
| B25.2 Cytomegaloviral pancreatitis |
| B25.8 Other cytomegaloviral diseases |
| B25.9 Cytomegaloviral disease, unspecified |
| B26.0 Mumps orchitis |
| B26.1 Mumps meningitis |
| B26.2 Mumps encephalitis |
| B26.3 Mumps pancreatitis |
| B26.8 Mumps with other complications |
| B26.8 (no description found) |
| B26.9 Mumps without complication |
| B27.0 Gammaherpesviral mononucleosis |
| B27.1 Cytomegaloviral mononucleosis |
| B27.8 Other infectious mononucleosis |
| B27.9 Infectious mononucleosis, unspecified |
| B30.0 Keratoconjunctivitis due to adenovirus |
| B30.1 Conjunctivitis due to adenovirus |
| B30.2 (no description found) |
| B30.3 Acute epidemic hemorrhagic conjunctivitis (enteroviral) |
| B30.8 Other viral conjunctivitis |
| B30.9 Viral conjunctivitis, unspecified |
| B33.0 Epidemic myalgia |
| B33.1 Ross River disease |
| B33.2 Viral carditis |
| B33.3 Retrovirus infections, not elsewhere classified |
| B33.4 Hantavirus (cardio-) pulmonary syndrome |
| B33.8 Other specified viral diseases |
| B34.1 Enterovirus infection, unspecified |
| B34.2 Coronavirus infection, unspecified site |
| B34.3 Parvovirus infection, unspecified site |
| B34.4 Papovavirus infection, unspecified |
| B34.8 Other viral infections of unspecified site |
| B34.9 Viral infection, unspecified |
| B97.0 Adenovirus as the cause of diseases classified to other chapters |
| B97.1 Enterovirus as the cause of diseases classified to other chapters |
| B97.2 Coronavirus as the cause of diseases classified to other chapters |
| B97.3 Retrovirus as the cause of diseases classified to other chapters |
| B97.4 Respiratory syncytial virus as the cause of diseases classified to other chapters |
| B97.5 Reovirus as the cause of diseases classified to other chapters |
| B97.6 Parvovirus as the cause of diseases classified to other chapters |
| B97.7 Papillomavirus as the cause of diseases classified to other chapters |
| B97.8 Other viral agents as the cause of diseases classified to other chapters |
| G02.0 Meningitis in viral diseases classified elsewhere |
| G05.1 Encephalitis, myelitis and encephalomyelitis in viral diseases classified elsewhere |
| H19.1 Herpesviral keratitis and keratoconjunctivitis |
| I41.1 Myocarditis in viral diseases classified elsewhere |
| J10.8 Influenza with other manifestations, influenza virus identified |
| J11 Influenza, virus not identified |
| J11.8 Influenza with other manifestations, virus not identified |
| M01.4 Rubella arthritis |
| M01.50 Arthritis in other viral diseases classified elsewhere, multiple sites |
| M01.51 Arthritis in other viral diseases classified elsewhere, shoulder region |
| M01.52 Arthritis in other viral diseases classified elsewhere, upper arm |
| M01.53 Arthritis in other viral diseases classified elsewhere, forearm |
| M01.54 Arthritis in other viral diseases classified elsewhere, hand |
| M01.55 Arthritis in other viral diseases classified elsewhere, pelvic region and thigh |
| M01.56 Arthritis in other viral diseases classified elsewhere, lower leg |
| M01.57 Arthritis in other viral diseases classified elsewhere, ankle and foot |
| M01.58 Arthritis in other viral diseases classified elsewhere, other site |
| M01.59 Arthritis in other viral diseases classified elsewhere, site unspecified |
| O98.4 Viral hepatitis complicating pregnancy, childbirth and the puerperium |
| O98.5 Other viral diseases complicating pregnancy, childbirth and the puerperium |
| P35.0 Congenital rubella syndrome |
| P35.1 Congenital cytomegalovirus infection |
| P35.2 Congenital herpesviral [herpes simples] infection |
| P35.3 Congenital viral hepatitis |
| P35.8 Other congenital viral diseases |
| P35.9 Congenital viral disease, unspecified |
| Z21 Asymptomatic human immunodeficiency virus [HIV] infection status |

**Influenza and pneumonia**

| J10.0 | Influenza due to identified seasonal influenza virus |
| --- | --- |
| J11.0 | Influenza, virus not identified |
| J12 | Viral pneumonia, not elsewhere classified |
| J13 | Pneumonia due to Streptococcus pneumoniae |
| J14 | Pneumonia due to Haemophilus influenzae |
| J15 | Bacterial pneumonia, not elsewhere classified |
| J16 | Pneumonia due to other infectious organisms, not elsewhere classified |
| J17 | Pneumonia in diseases classified elsewhere |
| J18 | Pneumonia, organism unspecified |

**Bronchiolitis**

| J21 | Acute bronchiolitis |
| --- | --- |
